# Interaction between chicken TRIM25 and MDA5 and their role in mediated antiviral activity against IBDV infection

**DOI:** 10.3389/fmicb.2022.1068328

**Published:** 2022-11-28

**Authors:** Elisabet Diaz-Beneitez, Liliana Lilibeth Cubas-Gaona, Oscar Candelas-Rivera, Ana Benito-Zafra, Maria Teresa Sánchez-Aparicio, Lisa Miorin, José F. Rodríguez, Adolfo García-Sastre, Dolores Rodríguez

**Affiliations:** ^1^Department of Molecular and Cellular Biology, Centro Nacional de Biotecnología, CSIC, Madrid, Spain; ^2^Department of Microbiology, Icahn School of Medicine at Mount Sinai, New York, NY, United States; ^3^Global Health and Emerging Pathogens Institute, Icahn School of Medicine at Mount Sinai, New York, NY, United States; ^4^Department of Medicine, Division of Infectious Diseases, Icahn School of Medicine, New York, NY, United States; ^5^The Tisch Cancer Institute, Icahn School of Medicine at Mount Sinai, New York, NY, United States; ^6^Department of Pathology, Molecular and Cell-Based MedicineI at Mount Sinai, Icahn School of Medicine, New York, NY, United States

**Keywords:** MDA5, TRIM25, IBDV, innate immunity, interferon

## Abstract

Infectious Bursal Disease Virus (IBDV) is the causative agent of an immunosuppressive disease that affects domestic chickens (*Gallus gallus*) severely affecting poultry industry worldwide. IBDV infection is characterized by a rapid depletion of the bursal B cell population by apoptosis and the atrophy of this chief lymphoid organ. Previous results from our laboratory have shown that exposure of infected cells to type I IFN leads to an exacerbated apoptosis, indicating an important role of IFN in IBDV pathogenesis. It has been described that recognition of the dsRNA IBDV genome by MDA5, the only known cytoplasmic pattern recognition receptor for viral RNA in chickens, leads to type I IFN production. Here, we confirm that TRIM25, an E3 ubiquitin ligase that leads to RIG-I activation in mammalian cells, significantly contributes to positively regulate MDA5-mediated activation of the IFN-inducing pathway in chicken DF-1 cells. Ectopic expression of chTRIM25 together with chMDA5 or a deletion mutant version exclusively harboring the CARD domains (chMDA5 2CARD) enhances IFN-β and NF-ĸB promoter activation. Using co-immunoprecipitation assays, we show that chMDA5 interacts with chTRIM25 through the CARD domains. Moreover, chTRIM25 co-localizes with both chMDA5 and chMDA5 2CARD, but not with chMDA5 mutant proteins partially or totally lacking these domains. On the other hand, ablation of endogenous chTRIM25 expression reduces chMDA5-induced IFN-β and NF-ĸB promoter activation. Interestingly, ectopic expression of either wild-type chTRIM25, or a mutant version (chTRIM25 C59S/C62S) lacking the E3 ubiquitin ligase activity, restores the co-stimulatory effect of chMDA5 in chTRIM25 knockout cells, suggesting that the E3-ubiquitin ligase activity of chTRIM25 is not required for its downstream IFN-β and NF-ĸB activating function. Also, IBDV-induced expression of *IFN-β*, *Mx* and *OAS* genes was reduced in chTRIM25 knockout as compared to wild-type cells, hence contributing to the enhancement of IBDV replication. Enhanced permissiveness to replication of other viruses, such as avian reovirus, Newcastle disease virus and vesicular stomatitis virus was also observed in chTRIM25 knockout cells. Additionally, chTRIM25 knockout also results in reduced MAVS-induced IFN-β promoter stimulation. Nonetheless, similarly to its mammalian counterpart, chTRIM25 overexpression in wild-type DF-1 cells causes the degradation of ectopically expressed chMAVS.

## Introduction

Innate immunity is the first line of defense against viral infection. While a strong innate immune response is required to combat virus infections and promote virus clearance, it is clear that this response must be tightly regulated to prevent undesirable activation on uninfected cells, as well as exacerbated responses and excessive inflammation during infection. Therefore, immune signaling pathways are highly regulated by numerous positive and negative interactions, many of which are targeted by viral proteins in order to facilitate virus replication. Innate immunity is characterized by the expression of type I interferons (IFNs) and other pro-inflammatory cytokines, which are produced when host pattern recognition receptors (PRRs) sense pathogen-associated molecular patterns (PAMPs). Among PRRs, toll-like receptors (TLRs) are associated to cellular membranes, and specifically TLR3 is present in endosomes where it recognizes viral RNA, while retinoic acid-inducible gene I (RIG-I)-like receptors (RLRs) are cytosolic PRRs. The RLRs family members RIG-I and melanoma differentiation-associated gene 5 (MDA5) become activated upon recognition of cytosolic viral RNAs, and this signal promotes the interaction with the mitochondrial antiviral-signaling protein (MAVS), that activates downstream interferon-regulatory factors 3 and 7 (IRF-3/7) and nuclear factor kappa-light-chain-enhancer of activated B cells (NF-ĸB) which translocate to the cell nucleus and induce type I IFN production. A third member of the RLR family is the laboratory of genetics and physiology 2 (LGP2), which lacks the N-terminal caspase activation and recruitment domain (CARD) and cannot activate *IFN-β* gene expression by itself, but can regulate RIG-I and MDA5-mediated antiviral signaling (Komuro and Horvath, 2006; Satoh et al., 2009). The secreted IFN stimulates the expression of a large number of genes whose products exert antiviral and immunomodulatory actions that can limit infection (Kato et al., 2005; Akira et al., 2006; Pichlmair et al., 2006).

RIG-I and MDA5 are structurally related, and both contain two N-terminal CARDs, responsible for downstream signaling transduction through their interaction with MAVS, a middle RNA helicase domain, and a C-terminal domain (CTD) (Saito et al., 2007; Cui et al., 2008) which recognizes and binds RNA. Structural differences in CTDs of RIG-I and MDA5 might explain their abilities to sense distinct viral RNAs. RIG-I was first reported to recognize short double-stranded RNA (dsRNA) structures and uncapped 5′-triphosphate (5′-ppp) as well as single-stranded RNA (ssRNA) generated by viral RNA polymerases (Hornung et al., 2006; Yoneyama and Fujita, 2009), while MDA5 has higher affinity for long dsRNA (Kato et al., 2006, 2008) and replicative intermediates of multiple positive-strand RNA viruses (Loo et al., 2008). Therefore, they are specialized in the recognition of different subsets of viruses, with RIG-I mainly sensing negative-strand RNA viruses such as Sendai virus (SeV), vesicular stomatitis virus (VSV), Newcastle disease virus (NDV) or influenza virus, while MDA5 appears to mostly detect infection of picornaviruses (Kato et al., 2006, 2008) and coronaviruses (Roth-Cross et al., 2008; Yin et al., 2021) although other viruses can also be recognized (Dias Junior et al., 2019).

The mammalian RLR signaling pathway has been extensively studied, and it is well established that activation of RIG-I and MDA5 takes place in a multistep process consisting of viral RNA binding, conformational changes and a series of posttranslational modifications, such as polyubiquitination, sumoylation and phosphorylation (Brisse and Ly, 2019). Proteins of the TRIM family, which are characterized by the presence of RING, B box, and coiled-coil domains, are involved in various cellular processes, such as cell proliferation, differentiation and death, and they are also implicated in innate immune signaling pathways by acting as E3 ubiquitin ligases (Rajsbaum et al., 2014). Specifically, TRIM25 has been shown to be required for RIG-I activation. In the absence of an RNA ligand, RIG-I is held in a closed conformation where the CARD domains are in association with the CTD (Saito et al., 2007). However, upon viral RNA recognition the RIG-I CARD domains are exposed and TRIM25 triggers the synthesis of Lysine 63 (K63)-linked polyubiquitin that binds to the N-terminal CARDs, leading to oligomerization and binding with MAVS *via* CARD-CARD interaction. As a consequence, subsequent signaling processes result in the production of type I IFN (Kawai et al., 2005; Gack et al., 2007; Chen et al., 2016). In addition, K63 ubiquitination in the RIG-I CTD mediated by the Riplet E3 ligase has also been shown to be required for RIG-I activation (Oshiumi et al., 2009, 2010). On the other hand, the presence of K63 ubiquitin modification on mammalian MDA5 is more controversial, with studies proposing that it is ubiquitinated (Lian et al., 2018) or not (Gack et al., 2007) by TRIM25.

Despite recent advances on the characterization of the chicken IFN system, our knowledge is still very limited as compared to the mammalian system. Notably, chickens lack RIG-I (Barber et al., 2010), but have an intact MDA5 that possesses a conserved domain architecture when compared with its mammalian counterpart (Karpala et al., 2011; Lee et al., 2012). Additionally, activation of chicken MDA5 (chMDA5) leads to the induction of type I IFN responses through its binding to the MAVS adaptor protein. Moreover, it has been shown that chMDA5 can functionally compensate the lack of RIG-I in the recognition of avian Influenza A virus (IAV) (Liniger et al., 2012; Rajsbaum et al., 2012), indicating that it plays a pivotal role in sensing RNA virus infections in chickens. On the other hand, chicken TRIM25 (chTRIM25) was identified and shown that, as its mammalian ortholog, it plays an important role in establishing an antiviral response against IAV in chicken cells, being required for virus-induced IFN-β production (Rajsbaum et al., 2012). chTRIM25 expression was shown to be upregulated in immune organs (spleen, thymus and Bursa of Fabricius) of NDV-infected chickens, as well as in chicken embryo fibroblast upon stimulation with the dsRNA mimic Poly I:C or infection with NDV (Feng et al., 2015). More recently it was described that chTRIM25 is also upregulated upon infection with avian leukosis virus (ALV) (Zhou et al., 2020), both in DF-1 fibroblasts and in chickens, and that it can upregulate the chMDA5 receptor-mediated type I interferon response. Upregulation of chTRIM25 was also observed in DF-1 and DT40 B lymphoid chicken cells infected with infectious bursal disease virus (IBDV) (Wang et al., 2021).

IBDV, the best-characterized member of the *Birnaviridae* family, is the etiological agent of an acute, highly contagious, and immunosuppressive disease known as IBD or Gumboro disease that causes major economic losses to the poultry industry worldwide (Van den Berg et al., 2000). IBDV infection mainly affects young domestic chickens (*Gallus gallus*), between 3 and 6 weeks of age, being immature IgM-bearing B lymphocytes present in the Bursa of Fabricius the primary target cells (Rodenberg et al., 1994). IBDV infection is characterized by a rapid depletion of the bursal B cell population and the atrophy of this chief lymphoid organ in developing chickens (Kim et al., 1998; Lam, 1998; Khatri and Sharma, 2006), where the maturation of lymphocyte population occurs (Ingrao et al., 2013). Due to the specific damage of immune cells, surviving chicks undergo severe immunosuppression, hence becoming highly susceptible to secondary infections and unresponsive to vaccination against other pathogens (Sharma et al., 2000).

IBDV is a non-enveloped virus, with a bi-partite dsRNA genome, consisting of segments A and B, of 3.2 and 2.8 kbp, respectively, which code for a total of five mature proteins (VP1 to VP5) (Dobos et al., 1979). Segment A contains two partially overlapping open reading frames, the first one encoding a nonstructural protein, VP5, while the second one codes for three proteins synthesized as a polyprotein precursor. The viral polyprotein is cotranslationally processed by the viral protease VP4 (Birghan et al., 2000; Feldman et al., 2006) to release the pVP2, VP3, and VP4 proteins, of which pVP2 undergoes a subsequent self-proteolytic event to render the mature VP2 protein (Irigoyen et al., 2009). Segment B contains a single open reading frame encoding VP1, the RNA-dependent RNA polymerase (Von Einem et al., 2004). IBDV particles are naked icosahedrons made up by trimers of the VP2 capsid protein that contain in their interior the genome in the form of ribonucleoprotein (RNP) complexes, where the dsRNA is wrapped up by the VP3 protein and complexed with the polymerase VP1 (Luque et al., 2009). VP3 is a multifunction protein that acts as scaffolding protein during virion assembly (Maraver et al., 2003), regulates VP1-mediated RNA-dependent RNA replication (Garriga et al., 2007; Ferrero et al., 2015), and due to its dsRNA binding capacity inhibits PKR-mediated apoptosis (Busnadiego et al., 2012) and antagonizes the innate immune response (Ye et al., 2014). In addition, VP3 and VP4 have been associated with the attenuation of IFN-β expression by targeting cellular factors involved in the innate immune response (Li et al., 2013; Deng et al., 2021a,b, 2022).

Although the molecular basis for IBDV pathogenicity and the exact cause of clinical disease are still unknown, there is a growing body of evidence pointing to a role of innate immunity in IBDV pathogenesis. The burst of the innate immune response during the acute stage of the infection produces a so-called cytokine storm that will be decisive for the development of the infection (Ingrao et al., 2013). Multiple factors may contribute to B cell depletion in chickens, but exacerbated apoptosis of B cells and the induction of cytokine expression, were suggested as potential killing mechanisms in the context of IBDV infection (den Berg, 2000a). In this regard, previous results from our laboratory have shown that IFN contributes to exacerbate apoptosis in IBDV infected cells (Cubas-Gaona et al., 2018). Hence, a better understanding of the interactions of IBDV with avian innate immune responses is essential for the control of the disease.

Work by Lee and colleagues showed that IBDV genome is recognized by MDA5 in chicken fibroblasts and macrophages, and this is a starting point for the IBDV-induced MDA5 signaling pathway, leading to IFN production (Lee et al., 2014; Lee C. C. et al.,2015). Moreover, they also showed that silencing of *chMDA5* gene expression during IBDV infection results in an enhancement of IBDV replication, while chMDA5 overexpression leads to a reduction in IBDV virus yields. However, it has also been reported that the VP3 viral protein blocks the binding of MDA5 to IBDV genomic dsRNA, inhibiting IFN-β induction (Ye et al., 2014). These results indicate that MDA5 senses IBDV infection in chicken cells to initiate innate immunity, which then activates an adaptive immune response and limits IBDV replication.

The regulation of MDA5-mediated signaling pathway in chicken cells has not been clearly defined as yet. Our aim was to study the potential regulatory role of TRIM25 on the MDA5 signaling pathway in chicken cells, and its contribution to control IBDV infection. This work will contribute to understanding the chicken innate immune response and will provide important information about the interplay between IBDV and essential players of the innate immune signaling pathway.

## Materials and methods

### Cells, viruses and infections

DF-1 (Chicken embryonic fibroblasts; ATCC CRL-12203) cells were grown in Dulbecco’s modified minimal essential medium (DMEM) supplemented with penicillin (100 U/ml), streptomycin (100 U/ml), gentamicin (50 μg/ml), fungizone (12 μg/ml), nonessential amino acids, and 10% fetal calf serum (FCS; Sigma-Aldrich). Transient transfections were performed with Lipofectamine 2000 (Invitrogen), Lipofectamine RNAiMAX (Invitrogen) or Fugene HD (Promega) following the manufacturer’s instructions.

IBDV infections were performed on preconfluent (<80%) cell monolayers with the Soroa strain, a cell-adapted serotype 1 virus, diluted in DMEM at a multiplicity of infection (MOI) of 2 PFU/cell, unless otherwise stated. After 1 h of adsorption at 37°C, the medium was removed and replaced with fresh DMEM supplemented with 2% FCS. Infected cells were incubated at 37°C until the specified times post-infection (pi).

Infections with Newcastle disease virus (NDV) that expresses the green fluorescence protein (GFP), NDV-GFP (Park et al., 2003), and vesicular stomatitis virus (VSV) expressing GFP, VSV-GFP (Ostertag et al., 2007), were performed as described above on preconfluent (<80%) DF-1 cell monolayers at MOIs of 1 and 0.1 PFU/cell. Infections with avian reovirus (ARV) strain S1133 (kindly provided by Dr. José Manuel Martínez Costas, CIQUS, Universidad de Santiago de Compostela. Spain) were performed at 2 PFU/cell.

### Antibodies

The following antibodies were used in immunofluorescence, Western blot or immunoprecipitation analyses: anti-HA monoclonal antibody (mAb; Cell Signaling, #3724), anti-Flag rabbit polyclonal antibody (Sigma, #F7425), anti-V5 mAb (Invitrogen, #R960), anti-β-actin mAb (Santa Cruz Biotechnology, #sc-47,778), and a polyclonal serum against the IBDV VP3 protein (Fernandez-Arias et al., 1998). In addition, anti-chTRIM25 antibodies were generated for this study by immunizing animals with a synthetic peptide (produced at ProteoGenix, France). A polyclonal serum anti-muNS protein (kindly provided by Dr. José Manuel Martínez Costas, CIQUS, Universidad de Santiago de Compostela. Spain) has been previously described (Touris-Otero et al., 2004).

### Virus titration

Supernatants from cultures infected with IBDV were collected and subjected to low-speed centrifugation (1,500×g for 5 min) to remove cell debris. The clarified cell supernatants were used to determine the extracellular virus titers by plaque assay using semisolid agar overlays followed by immunostaining as previously described (Méndez et al., 2015). GFP expression levels were used as readout of NDV-GFP and VSV-GFP virus replication. For this, cells were collected at 24 h pi, subjected to low-speed centrifugation, and cell pellets were resuspended in PBS. Cell samples were loaded in a 96-well black polystyrene microplate (flat bottom clear; Costar) and GFP was determined in an automate Spectramax iD3 microplate reader (Molecular Devices).

### Plasmid construction

To clone the *chMDA5* gene into an expression plasmid we extracted RNA from DF-1 cells grown in 6-well plates that were treated with Poly I:C (1 μg/ml; InvivoGen) for 24 h. Total RNA was extracted by using the High pure RNA isolation kit (Roche) according to the manufacturer’s instructions. Purified RNA (500 ng) was reverse transcribed into cDNA using random primers (ThermoFisher Scientific) and SuperScript III (Invitrogen) reverse transcriptase, according to the manufacturer’s protocol. The cDNA was amplified by PCR using Platinum Taq DNA Polymerase (Invitrogen) and the *chMDA5* gene-specific primers 5’TATAATCGATATGTACCCTTATGCCATGTCGGAGGAGTGCCGAGACGA3’ and 5’TATAGCTAGCTTAATCTTCATCACTTGAAGGACAATGAGATGC3´. Reactions were performed as follows: 2 min at 94°C; 30 cycles of 30 s at 94°C, 30 s at 60°C and 3 min at 72°C; and finally, 5 min at 95°C. The resulting DNA fragment was subcloned into the pCAGGS expression vector (Niwa et al., 1991). The forward primer includes a *Cla*I site and HA-tag sequence, and the reverse primer includes a *Nhe*I site. The plasmid was sequenced to assess the identity of the cloned fragment with the original sequence, and it was found that it had a 312 pb deletion that affected to both CARD domains (nucleotide positions 132–444). This plasmid was then named pCAGGS-HA-chMDA5 Short. To obtain a full version of the *chMDA5* gene, a DNA fragment corresponding to the deleted sequence, with *Cla*I and *Kpn*I sites at 5′ and 3′ end respectively, was chemically synthesized (GenScript). The DNA fragment was excised from the plasmid provided by manufacturer by digestion with *Cla*I and *Kpn*I restriction enzymes and subcloned into the pCAGGS-HA-chMDA5 Short vector digested with the same enzymes to reconstitute the entire *chMDA5* gene. The obtained plasmid was termed pCAGGS-HA-chMDA5 Full. Mutant versions of the *chMDA5* gene were generated by PCR using site-directed mutagenesis or overlapping PCR.

The pCAGGS-V5-chTRIM25 plasmid and pRK5-Flag-ubiquitin expressing plasmid have been previously described (Lim et al., 2005; Rajsbaum et al., 2012). In addition, we generated a mutant TRIM25 deficient in E3-ubiquitin ligase activity (pCAGGS-V5-chTRIM25 C59S/C62S). Specific site mutations were introduced by consecutive PCR using the pCAGGS-V5-chTRIM25 plasmid as template and two oligonucleotide primer pairs. The first PCR was performed with oligonucleotides F1 (5´-CGCGTCTAGAGCCTCTGCTAACCATG-3′), corresponding to a sequence in the plasmid located upstream of the *TRIM25* gene, and R1 that contains 2 nucleotide substitutions (G to C in both cases) which will introduce mutations at positions 215 and 224 after the start codon of the gene (5′- GTCCGGGAAGGTGGTGCGGGACTGCGGGGAGCTG-3′). A second PCR was performed with oligonucleotides F2 (5´-GCCAGGTGCGGGACTTCAGCTCCCCGCAGTCCCGCAC-3′) that contains 2 nucleotide substitutions (C to G in both cases) and R2 (5´-CGCGGCTAGCTCGAGTTACCAG-3′) that hybridizes to the 3’end of the gene. Using these two PCR fragments as template, a third PCR was performed with primers F1 and R2 to amplify the whole gene incorporating the described mutations. The resulting fragment was digested with *XbaI* and *XhoI* and cloned into the pCAGGS-V5-chTRIM25 after removal of the wild type *chTRIM25* gene by digestion with the same restriction enzymes.

To generate the chNF-kB reporter plasmid, a DNA fragment containing five copies of the sequence corresponding to the binding site of chicken NF-ĸB (Kaiser and Mariani, 1999) was synthesized (GeneScript). This fragment was inserted into the pSI-Check2-hRluc-hNFκB-firefly plasmid (Zhou et al., 2018) to replace the binding site for the human NF-κB, which is followed by the firefly *luciferase* gene, by a sequence containing the chicken NF-κB binding site. For this, the synthesized DNA fragment was excised from the plasmid provided by the manufacturer by digestion with *Not*I y *Sac*II restriction enzymes and cloned into the Check2-hRluc-hNFκB-firefly plasmid (this plasmid was a gift from Qing Deng [Addgene plasmid #106979]) previously digested with the same enzymes. The resulting plasmid, named pSI-chNFκB-Luc, also contains the *Renilla luciferase* gene to allow normalization of firefly luciferase expression.

The plasmid expressing chMAVS (James et al., 2019) was kindly provided by Dr. Stephen Goodbourn.

All constructs were sequenced to verify their identity with the original sequences.

### Luciferase reporter assays

Pre-confluent (<80%) DF-1 cell monolayers were transfected in 24-well plates with either 100 ng of IFN-β reporter plasmid (pLUCTER) (King and Goodbourn, 1994) together with 30 ng of the *Renilla* luciferase plasmid (pRL-null; Promega) to normalize for transfection efficiency, or with 50 ng of the NF-κB reporter plasmid (pSI-chNFκB-Luc), in combination with the different plasmid constructs, as indicated in the results section, using Lipofectamine 2000 (Invitorgen) at a 1:2 ratio in Opti-MEM (Gibco) medium. 8 h later, cells were transfected with 250 ng of Poly I:C or 100 ng of IBDV dsRNA. After 16 h, cells were lysed and luciferase assays were performed with the dual-luciferase assay kit (Promega) according to the manufacturer’s instructions. Luciferase activity was recorded using an Appliskan luminometer (Thermo Scientific). Firefly luciferase values were normalized to *Renilla* values, and the fold induction was calculated as the ratio of samples transfected with inducing plasmid versus samples transfected with the empty plasmid.

### Western blot assay

Transfected or infected cells were lysed in Laemmli’s sample buffer (62.5 mM Tris–HCL [pH 6.8], 2% sodium dodecyl sulfate [SDS], 0.01% bromophenol blue, 10% glycerol, and 5% β-mercaptoethanol). Protein samples were subjected to 10% SDS-polyacrylamide gel electrophoresis (PAGE), followed by electroblotting onto nitrocellulose membranes (Bio-Rad). Membranes were incubated with blocking buffer (Tris-buffered saline [TBS] containing 0.05% Tween 20 [TBST] and 5% nonfat dry milk) for 30 min at room temperature and incubated at 4°C overnight with the corresponding primary antibodies diluted in blocking buffer. Thereafter, membranes were washed with TBST and incubated with either a goat anti-rabbit IgG-peroxidase (Sigma) or a goat anti-mouse IgG-peroxidase conjugate (Sigma), and immunoreactive bands were detected by an enhanced chemiluminescence (ECL) reaction (SuperSignal Thermo Scientific) and recorded using the ChemiDoc Touch Imaging System (BioRad). Relative protein band intensities were determined using the ImageJ Software.^1^

### Confocal immunofluorescence microscopy

DF-1 cells grown on glass coverslips were transfected with the different chMDA5 and chTRIM25 expression plasmids. At 24 h post-transfection (pt), cells were fixed with 4% paraformaldehyde (PFA) in PBS overnight at 4°C and processed for immunofluorescence using a standard protocol. After washing with PBS, cells were permeabilized with 0.5% Triton X-100 in PBS for 10 min, and then blocked with 20% FCS in PBS. Thereafter, cells were incubated with the indicated primary antibodies diluted in blocking solution in a humidified chamber for 1 h at 37°C. After this period, coverslips were washed with PBS and incubated with secondary antibodies conjugated to either Alexa Fluor 488 or 546. Coverslips were mounted on microscope slides using ProLong Gold anti-fade reagent (Invitrogen). Images were acquired with Leica TSC SP8 confocal laser with STED module for super-resolution.

### Immunoprecipitation (IP) assay

DF-1 cells were transfected in 6-well plates with 500 ng of the different plasmids using Fugene HD (Promega) at a 1:4 ratio in Opti-MEM (Gibco) medium. At 24 h pt with the different chMDA5 and chTRIM25 expression plasmids, cells were collected and lysed with lysis buffer (20 mM Tris pH 7.5, 80 mM NaCl, 20 mM EDTA, 1% Nonidet P-40) supplemented with a protease inhibitor cocktail (Roche Applied Science). Cell lysates were centrifuged and supernatants incubated with the indicated antibodies overnight at 4°C. Thereafter, samples were incubated with protein G beads (GenScript) at 4°C for 2 h. The immunoprecipitates were washed extensively with lysis buffer and eluted with Laemmli’s sample buffer by boiling for 5 min. Samples were analyzed by Western blot assay.

### Caspase 3/7 activity assay

Quantification of caspase activity was carried out by using the Caspase-Glo 3/7 assay kit (Promega). For this, DF-1 cell monolayers grown in 24-well plates were infected in duplicate. At 24 h pi, cells were harvested in medium and kept frozen until their analysis. 25 μl of the cell lysates under study was added to the same volume of Caspase-Glo 3/7 reagent in a 96-well plate. Plates were gently shaken and then incubated in the dark at room temperature for 1 h before the luciferase activity was recorded using an Appliskan luminometer (Thermo Scientific).

### Genome editing with CRISPR/Cas9

We used the Alt-R CRISPR-Cas9 system developed by Integrated DNA Technologies (IDT). Two sequence-specific CRISPR RNA (crRNA) for the *chTRIM25* gene were designed using the Breaking-Cas website.^2^ The selected crRNA sequences were TCAGTCGGAGCCGAATCTGTTG and CATCTTCGACGCCCCCGTGACGG. These crRNAs, the conserved transactivating crRNA (tracrRNA) required for the formation of the guide RNA (gRNA) and the Cas9 nuclease were purchased from IDT. Following the manufacturer’s protocol, tracrRNA and crRNA were combined, and resulting crRNA: tracrRNA duplexes were then mixed with Cas9 protein. DF-1 cells were transfected in suspension with the combination of crRNA:tracrRNA with the Cas9 using Lipofectamine RNAiMAX (Invitrogen) in Opti-MEM (Gibco) medium according to the manufacturer’s instructions, and then seeded in 6-well plates.

At 48 h pt, DF-1 cells monolayers were tripsinized, diluted and seeded in 100 mm dishes to obtain isolated clones. Individual clones were selected and grown and subsequently tested for the expression of chTRIM25 by Western blot using anti-chTRIM25 antibodies. Selected clones were further analyzed by amplifying a region of genomic DNA around the site of interest with the primers 5´-CTTTGTGGGCTTGAGGAAAA-3′ and 5´-CCAAGGCCACATACAGGACT-3′. Resulting PCR amplicons were sequenced to confirm the mutations introduced in the *TRIM25* gene.

### Quantitative PCR analysis

Total RNA was isolated by using the NucleoSpin RNA plus (Macherey-Nagel) according to the manufacturer’s instructions. Purified RNA (250 ng) was reverse transcribed into cDNA by using random primers (ThermoFisher Scientific) and SuperScript III (Invitrogen) reverse transcriptase, according to the manufacturer’s protocol. The cDNA was then subjected to qPCR using the gene-specific primers indicated in Table 1. qPCRs were performed in duplicate using Power SYBR green PCR master mix (ThermoFisher Scientific), according to the manufacturer’s protocol, on an Applied Biosystems 7500 real-time PCR system instrument. Reactions were performed as follows: 2 min at 50°C; 10 min at 95°C; 40 cycles of 15 s at 95°C and 1 min at 60°C; and finally, 15 s at 95°C, 1 min at 60°C, 30 s at 95°C, and 15 s at 60°C to build the melt curve. Gene expression levels were normalized to the *Glyceraldehyde-3-Phosphate Dehydrogenase* (*GAPDH*) gene, and the results were calculated as fold changes in gene expression relative to mock-infected cells by using the delta–delta *C_T_* (threshold cycle) method of analysis. Dilutions of plasmids containing the sequence amplified by each set of primers run in parallel were used to establish the corresponding standard curves.

**Table 1:** List of primers used for RT-qPCR assays.

Gene	Forward primer (5′-3′)	Reverse primer (5′-3′)
Chicken *GAPDH*	ATCAAGAGGGTAGTGAAGGCTGCT	TCAAAGGTGGAGGAATGGCTGTCA
Chicken *IFN-β*	ACCAGGATGCCAACTTCTCTTGGA	ATGGCTGCTTGCTTCTTGTCCTTG
Chicken *Mx*	TTCACGTCAATGTCCCAGCTTTGC	ATTGCTCAGGCGTTTACTTGCTCC
Chicken *OAS*	GCAGAAGAACTTTGTGAAGTGGC	TCGGCTTCAACATCTCCTTGTACC
Chicken *PKR*	ACGTGGGACATGATTGAGCCAAAG	TGATGTAGTCAACTGGAGGGAGCA
Chicken *FasR*	CATGGATCACATCAATGATTTGG	CCGAGTGCTTTGTCACATGAA
Chicken *IL6*	GAACGTCGAGTCTCTGTGCTACA	CACCATCTGCCGGATCGT
IBDV segment A	AAGGGCAGCTACGTCGATCTAC	TGGCAACTTCGTCTATGAAAGC

### Statistics

GraphPad Prism version 5.03 software (GraphPad Software, La Jolla, CA) was used to determine statistical significance, using the Student unpaired two-tailed *t* test.

## Results

### TRIM25 interacts with MDA5 in chicken cells and upregulates the MDA5-mediated transcriptional activation of IFN-β and NF-κB

**Figure 1:**
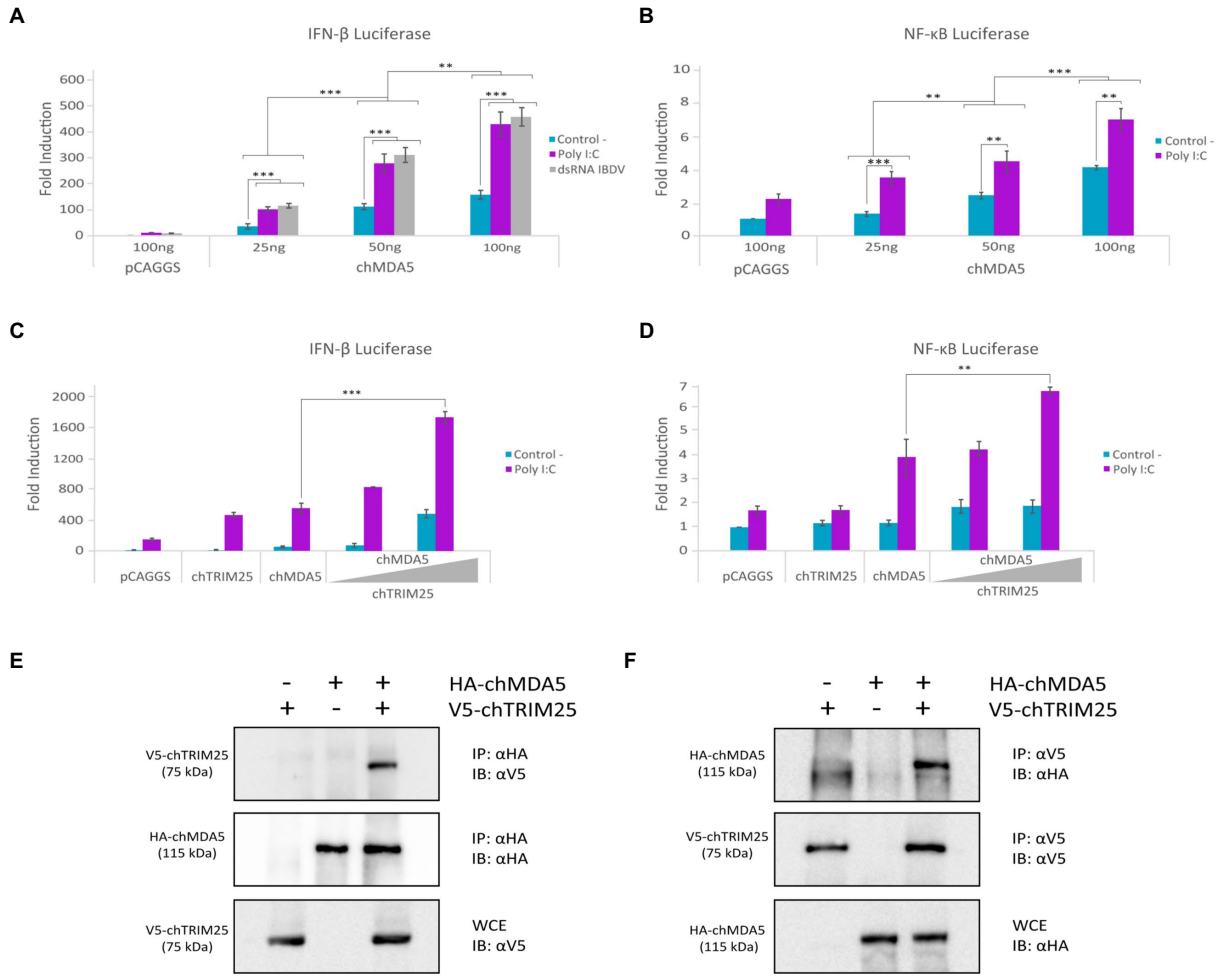
chTRIM25 is involved in chMDA5-mediated innate immune response. **(A)** DF-1 cells were co-transfected with different amounts (25, 50 or 100 ng) of HA-chMDA5 Full expression vector together with plasmids pLucter, carrying the firefly *luciferase* gene under the IFN-β promoter (100 ng), and pR-null, harboring the *Renilla luciferase* gene (30 ng). At 8 h pt, cultures were either mock transfected (control) or transfected with IBDV dsRNA (100 ng) or synthetic dsRNA (Poly I:C; 100 ng), and harvested 24 h after plasmid transfection. **(B)** DF-1 cells were co-transfected with different amounts of HA-chMDA5 Full expression vector as above together with the plasmid carrying the firefly *luciferase* gene under the NF-κB promoter (pSI-chNFκB-Luc; 50 ng). At 8 h pt, the cells were transfected with 250 ng of synthetic dsRNA (Poly I:C). Cultures were harvested 24 h after plasmid transfection. **(C)** DF-1 cells were transfected with pLucter (100 ng) and pR-null (30 ng) in combination with the plasmids expressing either V5-chTRIM25 (25 ng), HA-chMDA5 Full (25 ng), or a combination of the HA-chMDA5 Full (25 ng) plasmid with two amounts (25 or 50 ng) of the V5-chTRIM25 expression plasmid. At 8 h pt the cells were transfected with 250 ng of synthetic dsRNA (Poly I:C). Cultures were harvested 24 h after plasmid transfection. **(D)** DF-1 cells were transfected with pSI-chNFκB-Luc (50 ng) in combination with plasmids expressing either V5-chTRIM25 (25 ng), HA-chMDA5 Full (25 ng), or a combination of the HA-chMDA5 Full (25 ng) plasmid with two amounts (25 or 50 ng) of the chTRIM25 expression plasmid. At 8 h pt, the cells were transfected with 250 ng of synthetic dsRNA (Poly I:C). Cultures were harvested 24 h after plasmid transfection. A control consisting of cultures co-transfected with pLucter, pR-null, and the empty pCAGGS plasmid (100 ng) or with the pSI-chNFκB-Luc and empty pCAGGS plasmid (100 ng) was included in each assay. Cell extracts were analyzed using the dual luciferase assay kit, and each determination was carried out in duplicate. The firefly luciferase expression level of each sample was normalized using *Renilla* values. Bars indicate means ± standard derivations based on data of duplicate samples from three independent experiments. ** and *** indicate *p* values of <0.01 and < 0.001, respectively, as determined by unpaired Student’s test. **(E,F)** DF-1 cells were co-transfected with HA-chMDA5 Full and V5-chTRIM25 plasmids. At 24 h pt the cells were harvested and used for immunoprecipitation (IP) assays using specific antibodies against HA **(E)** or V5 **(F)**, followed by immnunoblotting (IB) with anti-V5 and anti-HA antibodies, respectively. Whole cell extracts (WCE) were analyzed by IB with anti-V5 **(E)** and anti-HA **(F)** antibodies, respectively.

With the aim of studying the regulation of chicken MDA5 (chMDA5)-mediated signaling pathway upon recognition of dsRNA in chicken fibroblast cells (DF-1), we first cloned the *chMDA5* gene in the pCAGGS expression vector and analyzed its ability to activate IFN-β and NF-κB responsive promoters using luciferase reporter assays. For this, chicken DF-1 cells were transfected with different amounts of the chMDA5 expression vector, together with either a plasmid carrying the *luciferase* gene under the IFN-β promoter (pLucter) or a similar plasmid in which the *luciferase* gene is downstream of the chicken NF-κB recognition sequence. At 8 h pt chMDA5 was activated by transfecting dsRNA, either viral (IBDV) or synthetic dsRNA (Poly I:C), and cells were harvested 16 h later. Figure 1A shows a chMDA5 dose-dependent activation of the IFN-β promoter, and how this activity was greatly enhanced in cells co-transfected with dsRNA. Comparable luciferase values were attained following transfection with either viral or synthetic dsRNA. Similarly, a dose-dependent response was observed on chMDA5-mediated NF-κB activity, which was also increased in the presence of dsRNA (Figure 1B).

It is well established that in mammalian cells TRIM25 interacts with the cytoplasmic receptor RIG-I, and regulates RIG-I-mediated signaling by the synthesis of K63-linked polyubiquitin (Gack et al., 2007). Despite the absence of RIG-I in chicken cells, previous results suggested that TRIM25 is important for virus-induced IFN-β production in chicken cells (Rajsbaum et al., 2012). Then, we considered of interest to explore whether TRIM25 could also regulate MDA5 activity in chicken cells. For this, DF-1 cells were transfected with the chMDA5 expressing plasmid and different amounts of a construct expressing chTRIM25, in the presence or absence of Poly I:C. Thereafter, IFN-β and NF-ĸB reporter assays were performed as described above. As shown in Figures 1C,D, a significant enhancement of both IFN-β and NF-κB activation was observed in cells co-transfected with increasing concentrations of the chTRIM25 plasmid upon activation with dsRNA with respect to cells transfected with either chMDA5 or chTRIM25 plasmids alone. These results indicated a possible collaboration between both proteins. Significantly, luciferase values remained nearly at basal levels in cells not transfected with dsRNA, even in the presence of chTRIM25, indicating that this collaboration would not override the requirement of dsRNA engagement for MDA5 activation.

To test whether chMDA5 interacts with chTRIM25, we performed a co-immunoprecipitation assay from DF-1 cells co-transfected with the plasmids expressing HA-tagged chMDA5 and V5-tagged chTRIM25 for 24 h. Immunoprecipitation was carried out using either anti-HA (Figure 1E) or anti-V5 (Figure 1F) antibodies. Figure 1E shows that the chTRIM25 protein co-precipitated with the chMDA5 protein when we used the anti-HA mAb as detected by immunoblot with anti-V5 mAb. Similarly, co-precipitation of chMDA5 was also obtained when immunoprecipitation was performed with the anti-V5 mAb (Figure 1F) as revealed by immunoblot assay with the anti-HA mAb. These results indicate an interaction between these two proteins.

### ChTRIM25-mediated upregulation of the transcriptional activation of IFN-β and NF-κB requires the chMDA5 CARD domains

**Figure 2:**
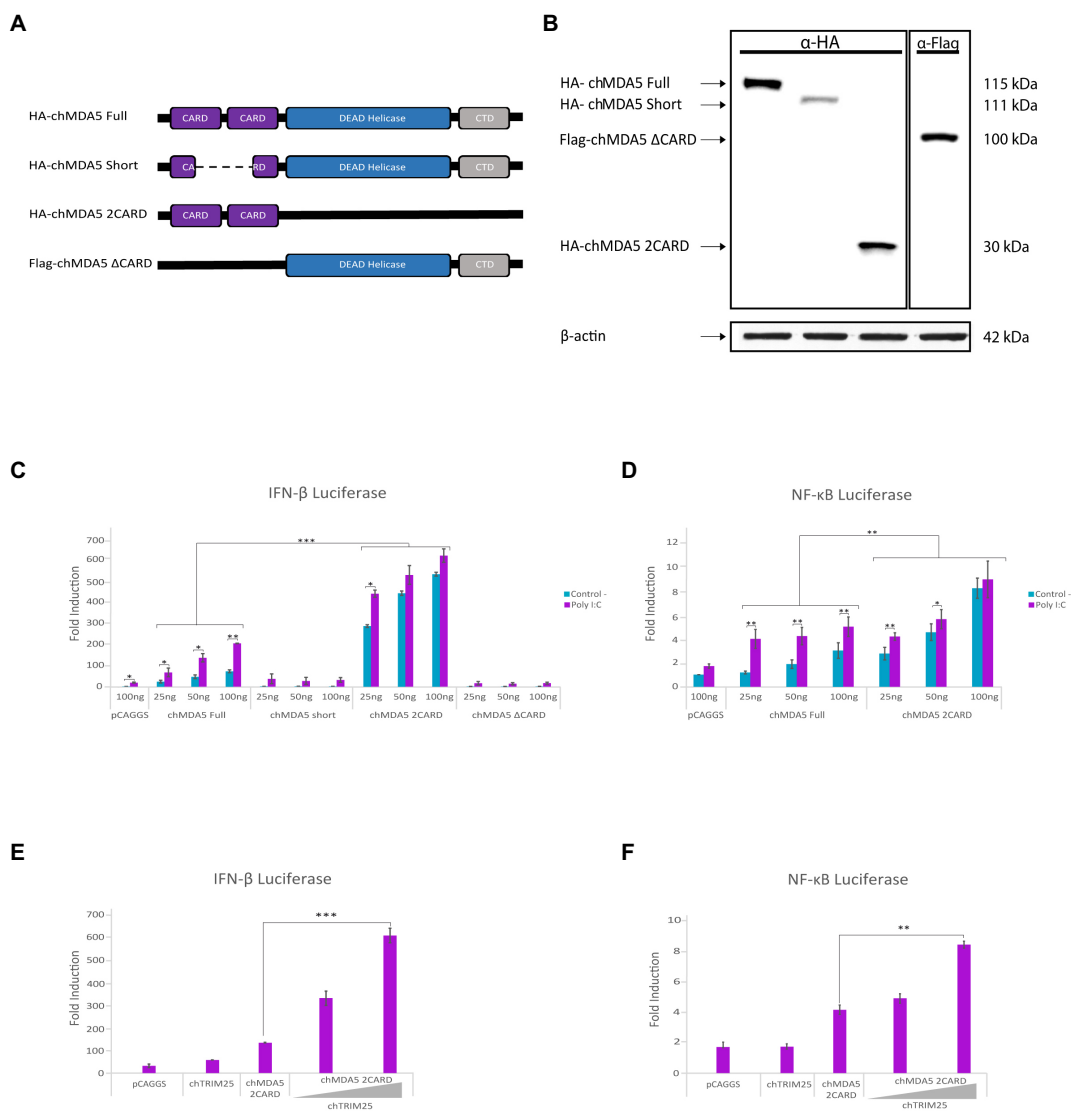
The CARD domains of chMDA5 are responsible for the activation of IFN-β and NF-κB promoters. **(A)** Schematic representation of the different HA or Flag tagged *chMDA5* gene versions used to assess the specific role of the chMDA5 domains: HA-chMDA5 Full carries the full-length chMDA5 coding sequence; HA-chMDA5 Short contains a deletion affecting both CARD domains; Flag-chMDA5 ∆CARD completely lacking both CARD domains; and HA-chMDA5 2CARD exclusively encoding both CARD domains. **(B)** DF-1 cultures transfected with plasmids expressing the different *chMDA5* gene versions were harvested at 24 h pt. Cell extracts were analyzed by Western blotting using anti-HA or anti-Flag antibodies, as indicated at the top of the panel. An antibody to β*-*actin was used for protein loading control. **(C,D)** DF-1 cultures were transfected with increasing amounts (25, 50 and 100 ng) of plasmids expressing the different versions of *chMDA5* gene together with the plasmids pLucter (100 ng) and pR-null (30 ng) expressing the firefly and *Renilla luciferase* genes, respectively **(C)** or with the plasmid pSI-chNFκB-Luc (50 ng) **(D)**. At 8 h pt, cultures were either mock transfected (control) or transfected with 250 ng of synthetic dsRNA (Poly I:C) and harvested at 24 h after plasmid transfection. **(E,F)** DF-1 cultures were co-transfected with pLucter (100 ng) and pR-null (30 ng) **(E)** or pSI-chNFκB-Luc (50 ng) **(F)**, along with plasmids expressing either V5-chTRIM25 (25 ng) or HA-chMDA5 2CARD (25 ng), or a combination of the HA-chMDA5 2CARD (25 ng) plasmid with two amounts (25 or 50 ng) of the V5-chTRIM25 expression plasmid. At 8 h pt the cells were transfected with 250 ng of synthetic dsRNA (Poly I:C) and cells were harvested at 24 h after plasmid transfection. A control consisting in cells co-transfected with pLucter, pR-null and the empty pCAGGS plasmid (100 ng) or with the pSI-chNFκB-Luc and empty pCAGGS plasmid (100 ng) was included in each assay. Samples were analyzed using the dual luciferase assay kit, and each determination was carried out in duplicate. The firefly luciferase expression level of each sample was normalized by *Renilla* values. Bars indicate means ± standard derivations based on data of duplicate samples from three independent experiments. *, ** and *** indicate *p* values of <0.05, <0.01 and < 0.001, respectively, as determined by unpaired Student’s test.

To determine which chMDA5 domain(s) is involved in the interaction with chTRIM25, we cloned into the pCAGGS plasmid two versions of the *chMDA5* gene, one lacking both CARD domains (pCAGGS-Flag-chMDA5 ∆CARD) and the other only containing both CARD domains (pCAGGS-HA-chMDA5 2CARD). In addition, we used the plasmid pCAGGS-HA-chMDA5 Short, that, as described in the material and methods section, carries a partially deleted chMDA5 sequence lacking part of both CARD domains, that was obtained by reverse transcription of mRNA from DF-1 cells (Figure 2A). The correct expression of these mutant proteins was confirmed by Western blot analysis of cells transfected with the different plasmids for 24 h, using specific antibodies against HA and Flag tags (Figure 2B). We then performed IFN-β or NF-ĸB reporter assays in DF-1 cells transfected with different amounts of these expression vectors, together with the reporter plasmids, to analyze the capacity of the different chMDA5 domains to activate IFN-β and NF-ĸB promoters in the absence or presence of dsRNA (Poly I:C). While, as shown before, the chMDA5 Full protein activates the IFN-β promoter in the presence of Poly I:C in a dose-dependent manner, neither chMDA5 Short nor the chMDA5 ∆CARD proteins were able to activate the IFN-β promoter under any of the tested conditions. However, transfection with the chMDA5 2CARD expression plasmid resulted in a considerable increase in the IFN-β promoter activation with respect to transfection with chMDA5 Full. This activation was dose-dependent but largely independent of Poly I:C (Figure 2C). Similar results were obtained with the NF-κB-Luc reporter plasmid. Figure 2D shows that both chMDA5 Full or chMDA5 2CARD were capable to induce NF-κB promoter activation, but again, while higher luciferase values were obtained upon dsRNA transfection in cells expressing chMDA5 Full, maximal IFN-β and NF-ĸB promoter activation was triggered by chMDA5 2CARD either in the absence or presence of dsRNA. These results, which indicate that the chMDA5 2CARD domain is constitutive active, are in agreement with previous studies showing that overexpression of this domain elicits a potent induction of the IFN-β promoter (Liniger et al., 2012; Cheng et al., 2015).

We then wished to elucidate the potential role of chTRIM25 on the activation of IFN-β and NF-ĸB promoter mediated by different chMDA5 domains. For this, DF-1 cells were co-transfected with a low dose of the plasmids expressing the different chMDA5 versions and increasing amounts of the plasmid expressing chTRIM25. As shown in Figure 2E, expression of chTRIM25 along with chMDA5 2CARD results in a significant enhancement of IFN-β promoter activation in a dose-dependent manner. A similar result was obtained with the NF-ĸB reporter plasmid (Figure 2F). However, when we analyzed the potential contribution of co-expression of chTRIM25 in combination with either chMDA5 Short or chMDA5 ∆CARD proteins we did not observe any cooperative effect (data not shown).

### The CARD domains of chMDA5 are involved in the interaction with chTRIM25

**Figure 3:**
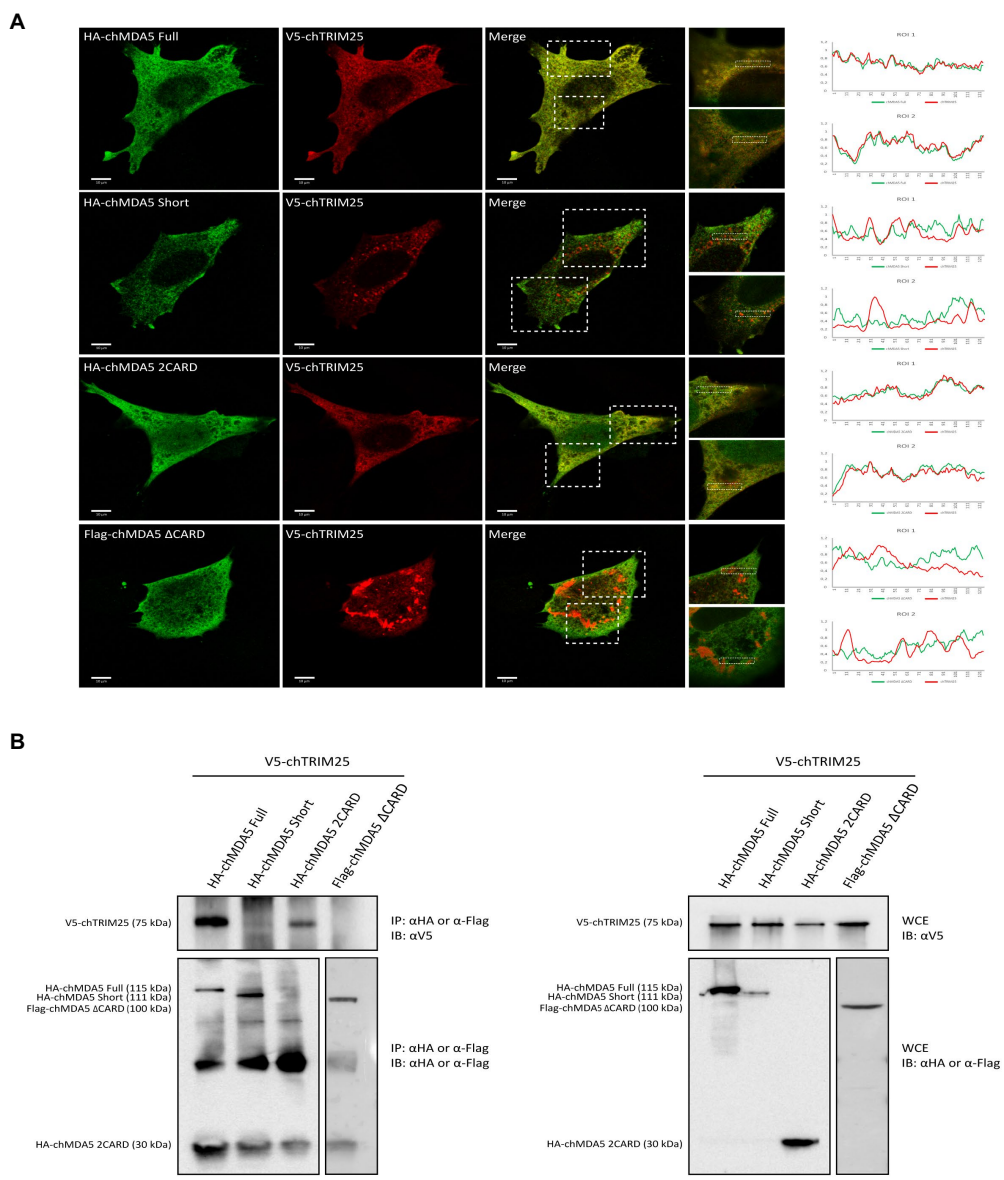
The CARD domains of MDA5 interact with TRIM25 in chicken cells. **(A)** DF-1 cells grown in glass coverslips were co-transfected with V5-chTRIM25 and HA-chMDA5 Full, HA-chMDA5 Short, HA-chMDA5 2CARD or Flag-chMDA5 ∆CARD versions. At 24 h pt, the cells were fixed with 4% paraformaldehyde and processed for immunofluorescence using antibodies against HA (Green), Flag (Green) and V5 (Red). Images were acquired with Leica TSC SP8 confocal laser with STED module for super-resolution. Insets show higher magnifications (x3) of boxed areas. The graphs at the right are histograms of the intensity of fluorescence profiles measured along the dashed line in the zoom. **(B)** Cells grown in regular 6-well plates were transfected as described above and harvested at 24 h pt. Collected cells were processed for immunoprecipitation (IP) using specific antibodies against HA or Flag followed by immunoblotting (IB) with anti-V5 antibodies (left panel). Whole cell extracts (WCE) were also analyzed by immunoblotting with specific antibodies against V5, HA or Flag.

Subsequently, we studied the subcellular distribution of the different versions of chMDA5 and chTRIM25 in DF-1 cells using immunofluorescence and confocal microscopy. DF-1 cells were co-transfected with the chTRIM25 plasmid and each of the plasmids carrying the different versions of chMDA5 for 24 h. As shown in Figure 3A, chMDA5 and its different deletion mutants show a diffuse distribution along the cell cytoplasm. A similar distribution pattern was observed for chTRIM25 in cells co-expressing either chMDA5 Full or chMDA5 2CARD. Moreover, higher magnification images of selected areas show an extensive co-localization of chTRIM25 and either chMDA5 Full or HA-chMDA5 2CARD. Also, the fluorescence intensity profiles show an almost complete overlap between chMAD5 Full or chMDA5 2CARD and chTRIM25. However, the distribution of chTRIM25 changes dramatically in cells co-expressing chMDA5 Short or chMDA5 ∆CARD, acquiring a dotted or granular pattern. The observation of higher magnification images highlights the differential distribution of chTRIM25 and the two chMDA5 mutants harboring CARD domain deletions. In addition, the intensity profiles show a slight overlap between the chTRIM25 and chMDA5 Short in certain areas, while completely different profiles were attained for chTRIM25 and chMDA5 ∆CARD.

Using co-immunoprecipitation studies, we have shown an interaction between chMDA5 and chTRIM25 (Figures 1E,F). In this regard, results described above point to the chMDA5 CARD domains as the regions engaged in the interaction with chTRIM25. To test this possibility, we performed co-immunoprecipitation assays using extracts from DF-1 cells co-transfected with the chTRIM25 expression vector and plasmids expressing the different chMDA5 protein versions. The efficient expression of HA-tagged chMDA5 Full, chMDA5 Short and chMDA5 2CARD or Flag-tagged chMDA5 ∆CARD proteins, and of V5-chTRIM15 was assessed by Western blot analysis of extracts from transfected cells (Figure 3B, right panel). As shown in Figure 3B (left panel), chMDA5 protein versions were efficiently immunoprecipitated, although in the case of chMDA5 2CARD the corresponding band cannot be distinguished from that of the immunoglobulin light chain since both proteins have the same molecular mass. Significantly, as observed in the upper part of the figure, chTRIM25 co-precipitated with both, chMDA5 Full and chMDA5 2CARD, but not with chMDA5 Short and chMDA5 ∆CARD. Hence, the co-immunoprecipitation assay demonstrated that chMDA5 interacts with chTRIM25 through the CARD domains.

### The E3-ubiquitin ligase activity of chTRIM25 is not required for its collaboration with chMDA5 in the IFN-β and NF-ĸB promoter activation

**Figure 4:**
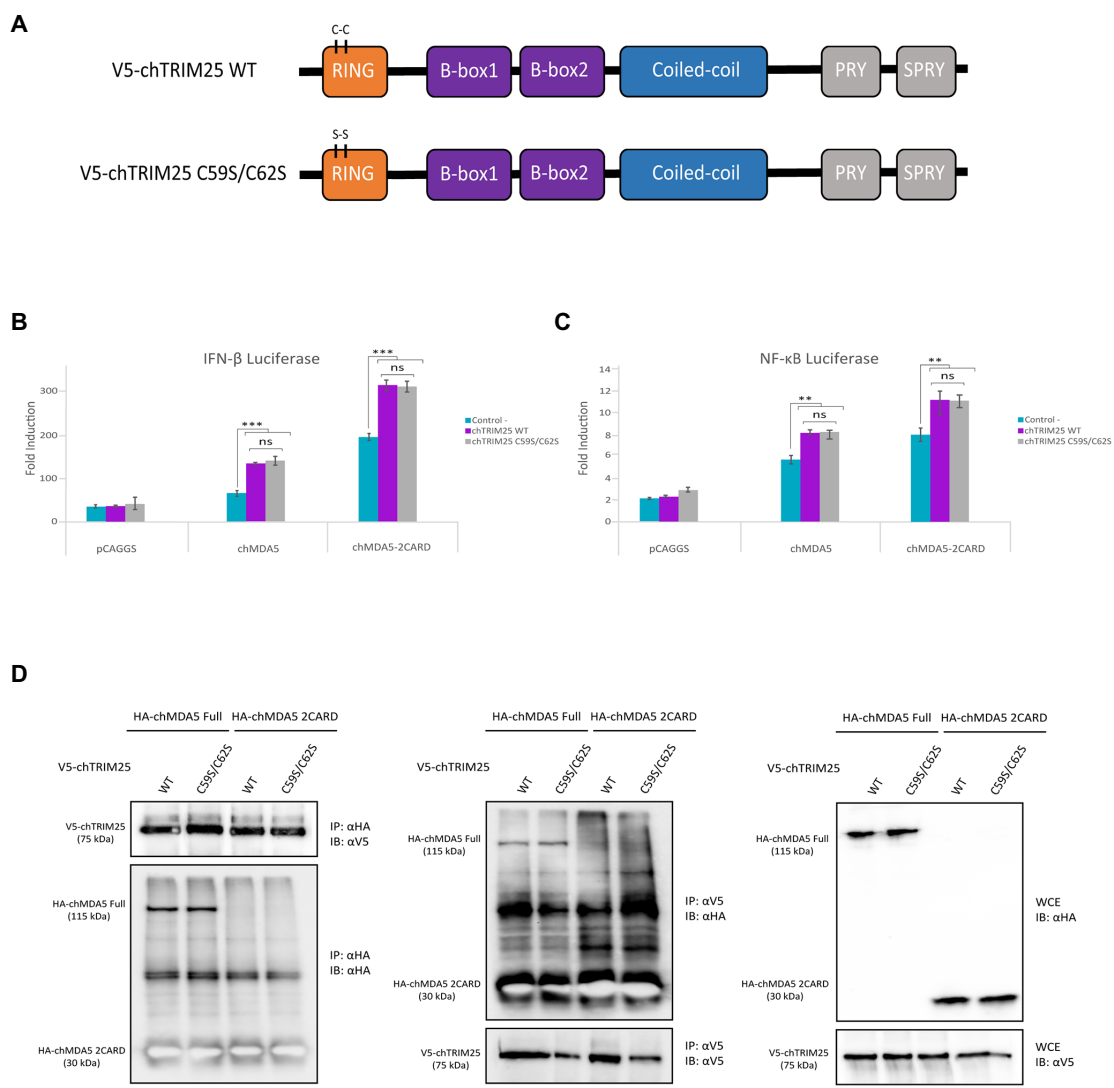
The E3-ubiquitin ligase function of chTRIM25 is neither required for IFN-β and NF-κB promoter activation nor for the interaction with chMDA5. **(A)** Schematic representation of the wild-type and the C59S/C62S mutant chTRIM25 proteins. **(B,C)** DF-1 cultures were co-transfected with HA-chMDA5 Full (25 ng) or HA-chMDA2 2CARD (10 ng) expression vectors together with plasmids expressing either the wild-type or the C59S/C62S mutant chTRIM25 proteins (25 ng), or with the empty pCAGGS plasmid, in combination with **(B)** pLucter and pR-null, or **(C)** pSI-chNFκB-Luc. At 8 h pt. the cells were transfected with 250 ng of synthetic dsRNA (Poly I:C). Cultures were harvested 24 h after plasmid transfection. A control consisting in cells co-transfected with pLucter, pR-null and the empty pCAGGS plasmid (50 ng) or with the pSI-chNFκB-Luc and the empty pCAGGS plasmid (50 ng) was included in each assay. Samples were analyzed using the dual luciferase assay kit, and each determination was carried out in duplicate. The firefly luciferase expression level of each sample was normalized using *Renilla* values. Bars indicate means ± standard derivations based on data for duplicate samples from three independent experiments. ** and *** indicate *p* values of <0.01 and < 0.001, respectively, as determined by unpaired Student’s test. ns, not significant. **(D)** DF-1 cells were co-transfected with either V5-chTRIM25 or V5-chTRIM25 C59S/C62S plasmids in combination with HA-chMDA5 Full or HA-chMDA5 2CARD. At 24 h pt the cells were collected and processed for immunoprecipitation (IP) using specific antibodies against HA followed by immunoblotting (IB) with anti-V5 and anti-HA antibodies (left panel), or immunoprecipitation with antibodies against V5 followed by immunoblotting with anti-HA and anti-V5 antibodies (middle panel). Whole cell extracts (WCE) were also analyzed by immunoblotting with specific antibodies against HA or V5 (right panel).

As mentioned before, it is well established that in mammalian cells TRIM25 regulates RIG-I signaling by mediating the K63-linked polyubiquitination of the CARD domains, thereby enabling an efficient interaction with the MAVS protein (Gack et al., 2007). Hence, we decided to examine whether the E3-ubiquitin ligase activity of chTRIM25 was required for the enhancement of chMDA5-mediated IFN-β and NF-ĸB promoter activation. For this, we generated a mutant version of chTRIM25 lacking the E3-ubiquitin ligase activity. As schematically represented in Figure 4A, the predicted chTRIM25 protein sequence shares conserved structural features with its mammalian counterpart. Specifically, the RING finger domain of chTRIM25 conserves the characteristic signature motif of E3 ubiquitin ligases (Joazeiro and Weissman, 2000; Meroni and Diez-Roux, 2005; Gack et al., 2007), and it has been recently shown that this domain is critical for the ubiquitin ligase activity in chicken cells (Wang et al., 2021). Therefore, we generated a new construct in which we introduced two amino acid substitutions, C59S and C62S, within the RING domain of chTRIM25 (chTRIM25 C59S/C62S). Similar changes in human TRIM25 were shown to abrogate its E3-ubiquitin ligase activity (Lee N. R. et al., 2015).

To carry out this study we used IFN-β and NF-ĸB reporter assays in DF-1 cells co-transfected with chMDA5 Full or chMDA5 2CARD in combination with wild-type chTRIM25 or chTRIM25 C59S/C62S. The activation of both, IFN-β and NF-ĸB promoters through the MDA5-or MDA5 2CARD-mediated signaling pathway observed in cells co-transfected with the mutant chTRIM25 C59S/C62S were similar to those detected in cells transfected with wild-type chTRIM25 (Figures 4B,C). This result suggests that the E3-ubiquitin ligase activity of chTRIM25 is not required for its downstream IFN-β and NF-ĸB activating function.

The next step was to perform co-immunoprecipitation assays to analyze the potential involvement of the chTRIM25 ubiquitinating function on the physical interaction with chMDA5. First, we confirmed the correct expression of the different proteins tested in this assay by Western blot analysis of whole cell extracts from transfected cells (Figure 4D, right panel). In addition, we showed that both chTRIM25 versions are able to co-precipitate with overexpressed chMDA5 or chMDA5 2CARD in DF-1 cells (Figure 4D, left panel). Similarly, chMDA5 and chMDA5 2CARD co-precipitated with both, the wild-type and the mutant version of chTRIM25 (Figure 4D, medium panel), although, as mentioned before, the immunoprecipitated chMDA5 2CARD protein could not be distinguished from the immunoglobulin light chain due their similar molecular mass.

### ChTRIM25 plays an essential role in chMDA5 signaling activation

**Figure 5:**
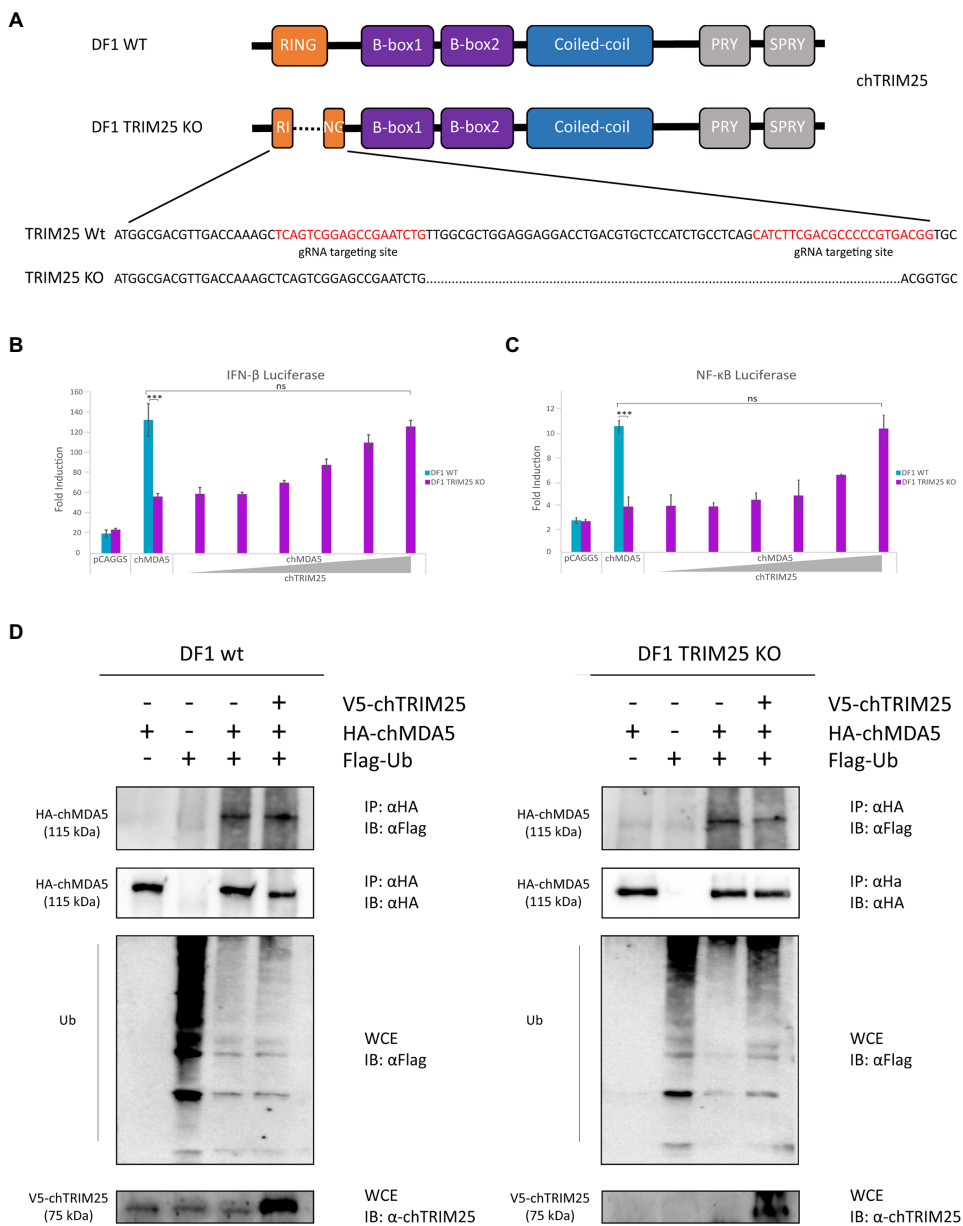
TRIM25 is essential for MDA5 signaling activation in chicken cells but it is not required for its ubiquitination. **(A)** Schematic representation of the structure of the *chTRIM25* gene and location of the gRNA targeting sites used to generate DF-1 TRIM25 KO cells. **(B,C)** DF-1 and DF-1 TRIM25 KO cells were co-transfected with **(B)** pLucter (100 ng) and pR-null (30 ng) plasmids, or with **(C)** the pSI-chNFκB-Luc plasmid (50 ng) together with the plasmid expressing HA-chMDA5 Full (25 ng) alone or in combination with different amounts (25, 50, 100, 200, 400 and 800 ng) of the V5-chTRIM25 expression vector. At 8 h pt, the cells were transfected with 250 ng of synthetic dsRNA (Poly I:C). Cultures were harvested 24 h after plasmid transfection. A control consisting in cells co-transfected with pLucter, pR-null and the empty pCAGGS plasmid (825 ng) or with the pSI-chNFκB-Luc and the empty pCAGGS plasmid (825 ng) was included in each assay. Samples were analyzed using the dual luciferase assay kit, and each determination was carried out in duplicate. The firefly luciferase expression level of each sample was normalized by *Renilla* values. Bars indicate means ± standard derivations based on data for duplicate samples from three independent experiments. *** indicates a *p* value of <0.001, as determined by unpaired Student’s test. ns, not significant. **(D)** DF-1 and DF-1 TRIM25 KO cells were co-transfected with V5-chTRIM25, HA-chMDA5 Full and Flag-Ubiquitin expressing plasmids during 24 h and then analyzed by immunoprecipitation (IP) using specific antibodies against HA followed by immunoblotting (IB) with anti-Flag or anti-HA antibodies. Whole cell extracts (WCE) were also analyzed by immunoblotting with specific antibodies against Flag or chTRIM25.

To further examine the role of chTRIM25 in chMDA5 activation, we generated chTRIM25 deficient DF-1 cells using CRISPR-Cas9 editing technology (Figure 5A). The sequence analysis revealed a 60 nucleotides deletion within the RING domain of chTRIM25 in DF-1 knockout cells (DF-1 TRIM25 KO). Western blotting and RT-qPCR results showed that neither chTRIM25 protein nor mRNA could be detected in extracts from the DF-1 TRIM25 KO cell line (data not shown).

When DF-1 TRIM25 KO cells were transfected with chMDA5, we observed that the activation of the IFN-β and NF-κB promoters was significantly lower than that found in wild-type DF-1 cells. However, the impairment of IFN-β and NF-κB promoter activation caused by chTRIM25 knockout could be compensated and rescued by chTRIM25 overexpression (Figures 5B,C). Interestingly, in agreement with our pervious results, overexpression of chTRIM25 C59S/C62S mutant also restored IFN-β and NF-κB promoter activation (Supplementary Figure 1). Collectively, these results indicate that chTRIM25 plays an essential role in chMDA5 signaling activation induced by cytosolic RNA sensing, which seems to be independent of its ubiquitinating function.

Because chTRIM25 interacts with chMDA5, and this interaction seems important for activation of MDA5 signaling, we investigated how chTRIM25 promotes chMDA5 activation. Although, as just mentioned, our results suggested that the ubiquitinating role of chTRIM25 was not required to regulate chMDA5-mediated signaling, we wished to further confirm these findings using the DF-1 TRIM25 KO cell line. For this, wild-type DF-1 and DF-1 TRIM25 KO cells were co-transfected with plasmids expressing chMDA5 Full, Flag-Ubiquitin (Flag-Ub) and chTRIM25. 24 h later, cells were harvested and used for co-immunoprecipitation assays. As shown in Figure 5D, chMDA5 was similarly ubiquitinated in both cells lines. Moreover, overexpression of chTRIM25 did not increase the ubiquitination of the exogenously expressed chMDA5 in these cell lines. These results indicate that chMDA5 undergoes a robust ubiquitination, and that this modification is not mediated by chTRIM25.

### ChTRIM25 knockout cells show reduced innate immune response to IBDV infection and support higher IBDV replication

**Figure 6:**
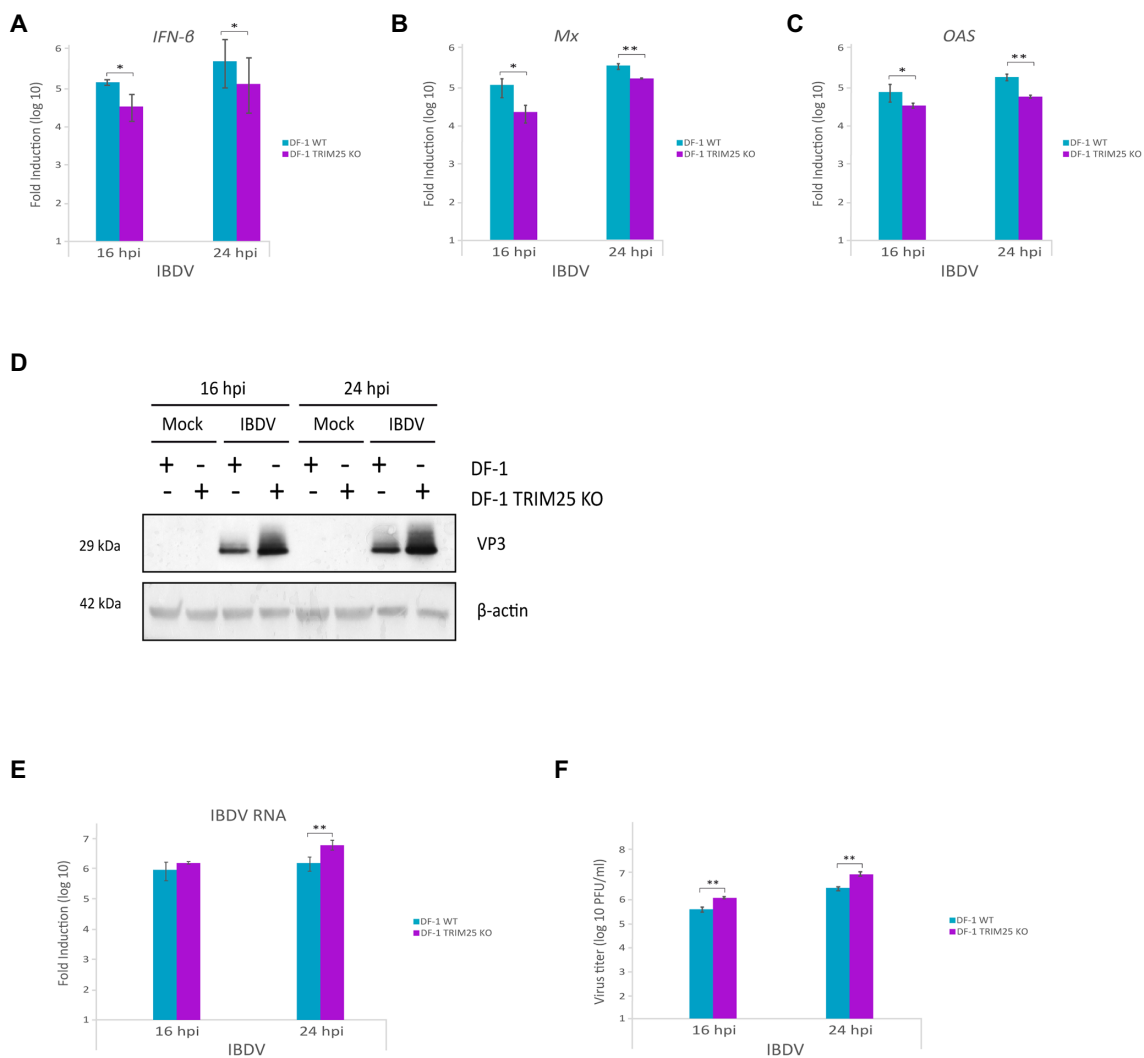
Downregulation of innate immune response by chTRIM25 knockout results in enhanced IBDV replication. DF-1 and DF-1 TRIM25 KO cells were mock-infected or infected with IBDV (MOI of 2 PFU/cell) and cells were harvested at 16 and 24 h pi for RNA extraction and RT-qPCR **(A–C,E)** or immunoblot **(D)** analyses. The expression levels of the selected genes, *IFN-β* **(A)**, *Mx* **(B)**, and *OAS* **(C)**, were determined by SYBR green-based RT-qPCR. Recorded values for each cellular gene were normalized to the *GAPDH* mRNA content and are presented on a log_10_ scale as the fold induction over the level found in mock-infected DF-1 or DF-1 TRIM25 KO cells. **(D)** Immunoblot analysis of total cell extracts with antibodies specifically recognizing the IBDV structural VP3 protein. Antibodies to β-actin were used for protein loading control. **(E)** The expression levels of the IBDV RNA were determined by SYBR green-based RT-qPCR. **(F)** Extracellular virus yields. Supernatants from infected cells were used for virus titration by a plaque assay. Bars indicate means ± standard derivations based on duplicate samples from three independent experiments. *, and ** indicate *p* values of <0.05 and < 0.01, respectively, as determined by unpaired Student’s test.

We next examined the role of chTRIM25 in host defense against IBDV infection. Previous studies have shown that chMDA5 is essential for the activation of the host innate immune response during IBDV infection (Lee et al., 2014; Lee C. C. et al., 2015). Then, to assess the effect of chTRIM25 knockout on the IFN response and IBDV infection we challenged DF-1 TRIM25 KO cells with IBDV. For this, DF-1 and DF-1 TRIM25 KO cell monolayers were infected at a MOI of 2 PFU/cell and samples were harvested at 16 and 24 h pi for different assays. Using RT-qPCR, we analyzed the expression levels of *IFN-β* as well as of those of different ISGs, i.e., *Mx*, *OAS*, *PKR*, *FasR* and *IL6*. Gathered data demonstrated that IBDV infection induces *IFN-β* gene expression in DF-1 cells. However, a significant reduction in *IFN-β* gene expression was observed in TRIM25 KO-infected cell samples compared to that observed in parental wild-type cells, at both 16 and 24 h pi (Figure 6A). Similarly, lower *Mx* (Figure 6B) and *OAS* (Figure 6C) mRNA levels were detected in DF-1 TRIM25 KO cells as compared with the control cells. Reduced expression of *PKR*, *FasR* and the proinflammatory cytokine *IL6* mRNAs were also observed at 24 h pi, although, in this case, the differences were not statistically significant (data not shown).

We then examined whether chTRIM25 knockout affects IBDV replication and infectious virus yields. For this, we first analyzed the accumulation of the viral protein VP3 in wild-type DF-1 and DF-1 TRIM25 KO infected cells. Western blotting revealed that the accumulation of the VP3 polypeptide in samples from DF-1 TRIM25 KO cells was significantly higher than that detected in wild-type infected cells at both times pi (Figure 6D). We next compared the relative abundance of the IBDV RNA by RT-qPCR analysis. As shown in Figure 6E, accumulation of IBDV RNA in DF-1 TRIM25 KO samples was also higher than that found in wild-type infected cells. Similarly, higher IBDV titers were detected in supernatants from DF-1 TRIM25 KO-infected cells (Figure 6F).

We have previously described that apoptotic death of IBDV-infected cells was related with the upregulation of IFN-β expression (Cubas-Gaona et al., 2018). Then, we wished to determine whether the chTRIM25 knockout also affects apoptotic cell death of IBDV-infected cells. The analysis of caspase activity in extracts from cells infected as described above revealed an important reduction of apoptotic cell death in DF-1 TRIM25 KO as compared with wild-type cells (Supplementary Figure 2).

Overall, these results demonstrate that chTRIM25 is important for host defense against IBDV infection by mediating MDA5 activation and IFN-β production and reducing virus progeny yields in chicken cells.

### The chTRIM25 knockout also favors replication of NDV, VSV and avian reovirus

**Figure 7:**
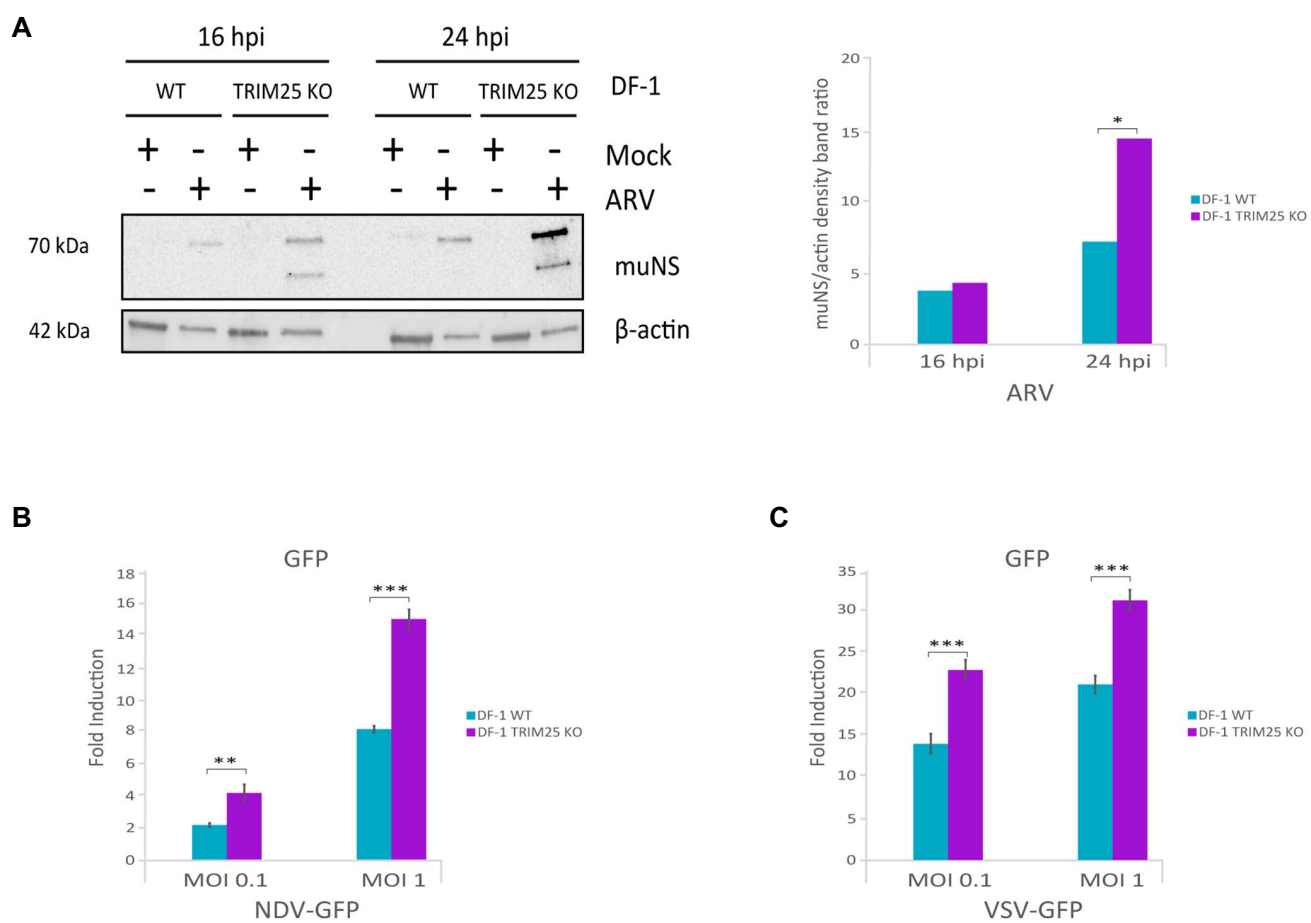
ChTRIM25 knockout also leads to increased ARV, NDV and VSV replication. **(A)** DF-1 and DF-1 TRIM25 KO cells were mock-infected or infected with ARV (MOI of 2 PFU/cell) and harvested at 16 and 24 h pi for immunoblot analysis of total cell extracts with antibodies specifically recognizing the ARV muNS protein (left panel). Antibodies to β-actin were used for protein loading control. The relative intensities of muNS were normalized to β-actin (right panel). **(B,C)** DF-1 and DF-1 TRIM25 KO cells were mock-infected or infected with recombinant NDV-GFP or VSV-GFP viruses (MOI of 0.1 and 1 PFU/cell, respectively) and cells were harvested at 24 h pi for GFP quantification. GPF levels were determined by analyzing extracts from cells infected with NDV-GFP **(B)** or VSV-GFP **(C)** using a Spectramax iD3 microplate reader. Bars indicate means ± standard derivations based on duplicate samples from three independent experiments. *, ** and *** indicate *p* values of <0.05, <0.01 and < 0.001, respectively, as determined by unpaired Student’s test.

The enhancement of IBDV replication upon chTRIM25 knockout is consistent with data showing the involvement of chTRIM25 on the upregulation of the MDA5-mediated innate immune response. Thus, it was interesting to investigate the potential role of chTRIM25 on the control of other viral infections. For this, we challenged wild-type DF-1 and DF-1 TRIM25 KO cells with another double-stranded RNA virus, i.e., avian reovirus (ARV), and two single-stranded RNA viruses, namely NDV and VSV. DF-1 cells were infected with ARV at a MOI of 2 PFU/cell, and at 16 and 24 h pi cells were collected for Western blot analysis using an antibody against the muNS protein. As shown in Figure 7A higher accumulation of both isosforms of the protein was observed in DF-1 TRIM25 KO cells when compared to wild-type DF-1 cells, at both pi times. For NDV and VSV infections, we used NDV-GFP and VSV-GFP recombinant viruses and infections were performed at MOIs of 0.1 and 1 PFU/cell and cells were harvested at 24 h pi. GPF levels, used as readout of virus replication, were significantly higher in DF-1 TRIM25 KO infected with either dose of NDV-GFP (Figure 7B) or VSV-GFP (Figure 7C) than those obtained in wild-type DF-1 cells. We then wished to determine whether the increase of viral replication in the DF-1 TRIM25 KO cells correlates with an attenuation of the innate immune response. However, RT-qPCR results showed very low *IFN-β*, *Mx*, *OAS* and *PKR* expression levels at the analyzed time pi in both wild-type and TRIM25 KO DF-1 cell lines infected with either NDV-GFP or VSV-GFP. Nonetheless, these results indeed indicate that abrogation of chTRIM25 expression results in higher permissiveness to viral replication.

### Role of chTRIM25 in MAVS-mediated signaling

**Figure 8:**
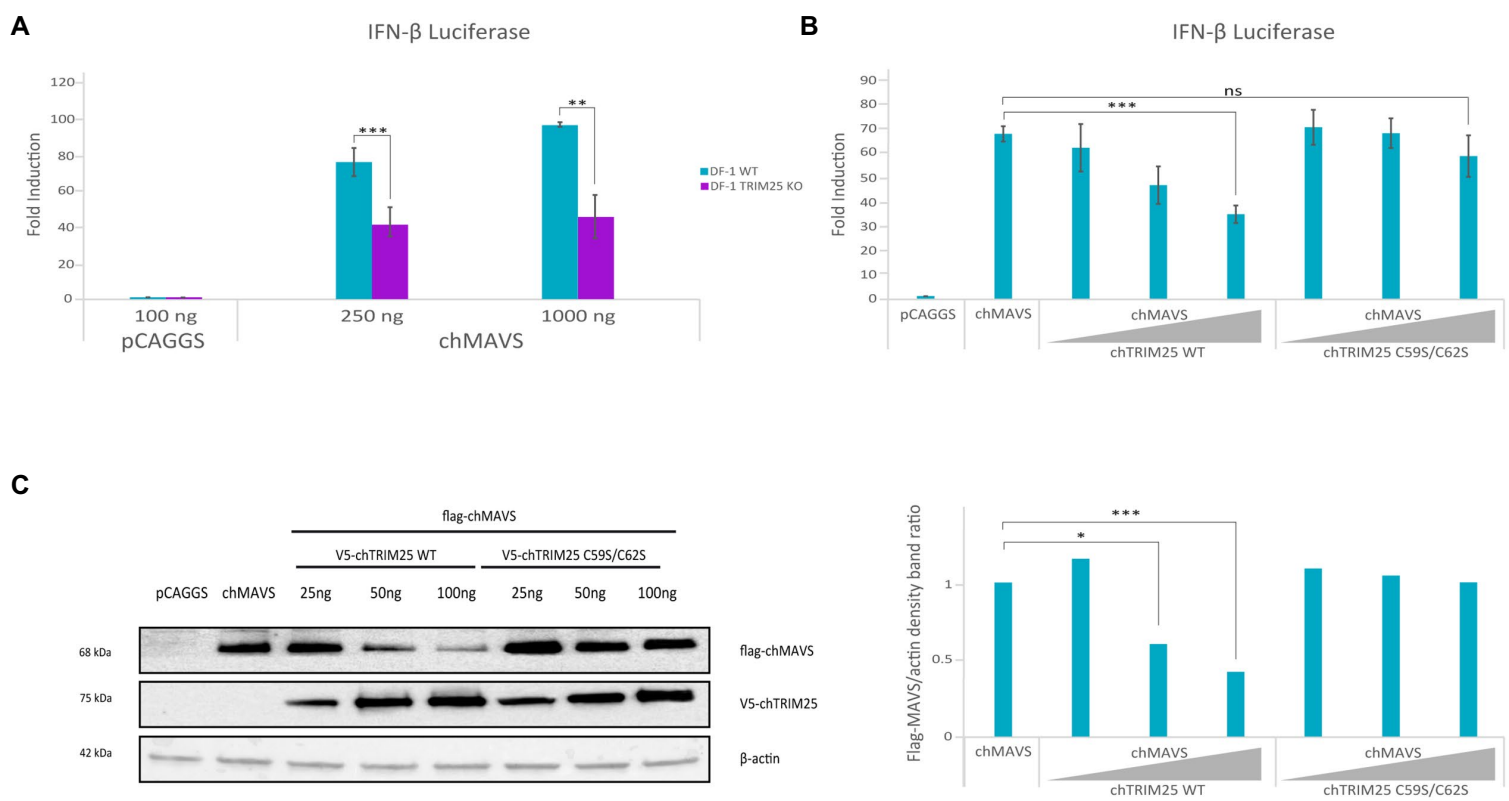
TRIM25 acts downstream of MAVS in the IFN-β induction pathway in chicken cells, and its overexpression is related with MAVS degradation. DF-1 and DF-1 TRIM25 KO cell cultures were co-transfected with pLucter (100 ng) and pR-null (30 ng) together with the Flag-chMAVS (250 or 1,000 ng) expression vector either alone **(A)**, or in combination with different amounts (25, 50 or 100 ng) of the plasmids expressing either the wild-type or the C59S/C62S mutant chTRIM25 proteins **(B,C)**. A control consisting in cells co-transfected with pLucter, pR-null and the empty pCAGGS plasmid (1000 or 350 ng) was included in each assay. **(A,B)** Samples were analyzed using the dual luciferase assay kit, and each determination was carried out in duplicate. The firefly luciferase expression level of each sample was normalized using *Renilla* values. **(C)** The expression levels of chMAVS and chTRIM25 in cells transfected as described in B were examined by immunoblot analysis using anti-Flag and anti-V5 antibodies, respectively (left panel). Antibodies to β-actin were used for protein loading control. The relative intensity of Flag-chMAVS was normalized to β-actin (right panel). Bars indicate means ± standard deviations based on data of duplicate samples from three independent experiments. ns, not significant. *, ** and *** indicate *p* values of <0.05, <0.01 and < 0.001, respectively, as determined by unpaired Student’s test.

As in mammalian cells, upon activation, chMDA5 associates with the downstream adaptor protein chMAVS (Liniger et al., 2012). We investigated the effect of chTRIM25 knockout in chMAVS induced IFN-β promoter activation to decipher at which step of the pathway does chTRIM25 act. Hence, DF-1 and DF-1 TRIM25 KO cells were transfected with two different doses of the chMAVS expression plasmid along with the IFN-β reporter plasmid. Data presented in Figure 8A show a significant reduction in the IFN-β promoter activity in DF-1 TRIM25 KO cells as compared with wild-type cells, indicating that chTRIM25 acts downstream of chMAVS. Interestingly, overexpression of chTRIM25, but not of chTRIM25 C59S/C62S mutant, in wild-type DF-1 cells produces a dose-dependent reduction of the IFN-β promoter activity upon activation of the pathway with MAVS (Figure 8B). The analysis of MAVS protein content in cells co-transfected with chMAVS and different doses of wild-type or mutant chTRIM25 by Western blot revealed a significant decrease in the amount of chMAVS in cells co-transfected with wild-type chTRIM25, in a dose-dependent manner. However, the presence of chMAVS was not affected by co-expression of the chTRIM25 C59S/C62S mutant (Figure 8C). In view of these results, we analyzed *MAVS* mRNA levels by RT-qPCR in these cells. Similar levels of *chMAVS* mRNA were detected in cells transfected with chMAVS alone or in combination with either wild-type or mutant chTRIM25 (data not shown). These results indicated that, as shown for its mammalian counterpart, chTRIM25 is involved in chMAVS degradation. Unfortunately, our attempts to determine if MAVS degradation occurs at the proteasome were unsuccessful, since treatments of DF-1 cells with various proteasome inhibitors were very cytotoxic.

## Discussion

The innate immune response, characterized by the production of IFN and other pro-inflammatory cytokines, plays a critical role as a first line of host defense. Nonetheless, a tight regulation of this powerful defense mechanism is crucial to prevent improper or exacerbated harmful activation that could cause immune damage to the host. While the regulation of the mammalian system has been extensively studied, much less attention has been paid to the chicken IFN system. In the absence of RIG-I in chicken cells, MDA5 is critical for recognition of cytoplasmic viral RNA. In this study we report that the E3 ubiquitin ligase TRIM25 positively regulates MDA5-mediated signaling in chicken cells, confirming previous results obtained by Zhou and colleagues in a recent study (2020). First, we show that chMDA5 triggers IFN-β and NF-kB promoter activation in the presence of dsRNA, either of viral (IBDV), or synthetic (Poly I:C) origin, in a dose dependent manner. Higher IFN-β and NF-kB promoter activation is achieved by overexpressing chMDA5 2CARD, a truncated version corresponding to the N-terminal moiety encompassing the CARD domains, and this activity is mostly independent of dsRNA. These results are in agreement with previous findings in both mammalian (Lee C. C. et al., 2015) and chicken cells (Liniger et al., 2012; Cheng et al., 2015). However, two other chMDA5 deletion mutants partially or totally devoid of the two CARD domains, chMDA5 Short and chMDA5 ∆CARD, respectively, are unable to activate the IFN signaling cascade. Significantly, co-expression of chTRIM25 together with chMDA5 or chMDA5 2CARD, but not with chMDA5 Short or chMDA5 ∆CARD, leads to an enhancement of IFN-β and NF-kB promoter activation. In accordance with this activation activity, it was observed that chTRIM25 interacts with chMDA5, and this interaction seems to be mediated by the MDA5 CARD domains, as suggested by both, co-precipitation and co-localization analyses. Interestingly, while chMDA5, and its different deletion mutants, exhibit a homogeneous cytoplasmic distribution independently of the presence or absence of ectopic chTRIM25, the distribution pattern of chTRIM25 drastically changes in cells co-expressing either chMDA5 Full or chMDA5 2CARD. In cells overexpressing TRIM25 alone, a dotted or granular pattern, reminiscent to the distribution described for the human TRIM25 (Sánchez-Aparicio et al., 2017), is observed. In contrast, in cells co-expressing chMDA5 Full or chMDA5 2CARD a homogeneous distribution of chTRIM25, similar to that of chMDA5, is detected. However, chTRIM25 retains its granular distribution pattern in cells co-expressing chMDA5 Short or chMDA5 ∆CARD. These results indicate that chMDA5 causes intracellular relocalization of chTRIM25 by its interaction through the CARD domains. On the other hand, co-transfection of dsRNA does not alter the observed distribution patterns of chTRIM25 and chMDA5 nor those of their different mutant versions (data not shown). Significantly, as mentioned above, a granular distribution of overexpressed human TRIM25 was also observed in mammalian cells, however, apparently this pattern was not altered in the presence of its interaction partner RIG-I, that showed mainly a homogeneous cytoplasmic distribution. Nonetheless, using bimolecular fluorescence complementation (BiFC) and super-resolution microscopy it was shown that TRIM25 and RIG-I colocalized in the dot-like structures, which were devoid of MAVS. In addition, it has been shown that TRIM25 granules were formed in NDV- (Yoo et al., 2014) and SeV-infected cells (Sánchez-Aparicio et al., 2017), where TRIM25 colocalized with stress granules markers. Hence, these results indicate that in mammalian cells TRIM25 is recruited into stress granules upon stimulation of the innate immune response ether by transfection-based plasmid overexpression or virus infection, where it interacts with RIG-I, but not with MAVS. It is tempting to speculate that chTRIM25 aggregates are also related to stress granules, however, upon interaction with chMDA5, and possibly with chMAVS, it is mobilized to their localizations to initiate the signaling cascade. Further studies will be required to test this hypothesis.

It is well established that K63-linked ubiquitination of RIG-I by TRIM25 (Gack et al., 2007) and Riplet (Oshiumi et al., 2013) is essential for RIG-I activation and downstream signaling. However, the role of K63-linked ubiquitin in MDA5 activation is still under debate. The chTRIM25 stimulatory effect on chMDA5-mediated signaling pathway described here could be related with its E3-ubiquitin ligase activity. In this regard, it was reported that TRIM25 contributes to MDA5 K63 ubiquitination in mammalian cells (Lian et al., 2018). With this in mind, we constructed a plasmid expressing a mutated version of chTRIM25 that harbors two amino acid changes, C59S and C62S, within the RING domain that, based on previous work with the human ortholog, should abrogate its enzymatic activity. Significantly, similar co-stimulatory effects on chMDA5- or chMDA5 2CARD-induced IFN-β and NF-kB promoter activation were obtained with the wild-type and the mutant versions of chTRIM25, suggesting that the E3-ubiquitin ligase activity of chTRIM25 is not required for its downstream IFN-β and NF-ĸB activating function. Moreover, the interaction with chMDA5 and chMDA5 2CARD, determined by co-precipitation analysis, is not affected by the mutations within the chTRIM25 RING domain. The successful inactivation of the E3-ubiquitin ligase activity in the chTRIM25 C59S/C62S mutant is supported by the results obtained following MAVS overexpression (Figure 8). To further investigate the role of chTRIM25 on the IFN-inducing pathway, a TRIM25 KO DF-1 cells line was generated by the CRISPR/cas9 technology. MDA5-mediated IFN-β and NF-kB promoter activation is significantly reduced in these cells as compared with the parental DF-1 cells. However, IFN-β and NF-kB promoter activation is successfully rescued in these cells by exogenous expression of recombinant wild-type chTRIM25 or mutant chTRIM25 C59S/C62S. Additionally, we also analyzed the potential ubiquitination of chMDA5 in parental and DF-1 TRIM25 KO cells. Our results indicate that chMDA5 is ubiquitinated, but this modification is not dependent on the presence of chTRIM25. Overall, these results indicate that chTRIM25 is an important regulator of chMDA5 activity, but this function does not seem to be related with the ubiquitination of chMDA5. A possibility likely explaining these findings is that the interaction with chTRIM25 might somehow promote the binding of the chMDA5-RNA complex (or chMDA5 2CARD) to MAVS. However, at present we do not have experimental data to support this hypothesis. On the other hand, our results indicate that another E3-ubiquitin ligase should be responsible for chMDA5 ubiquitination. In this regard, Lang and co-coworkers (Lang et al., 2017) reported that in mammalian cells another TRIM protein, TRIM65, interacts with MDA5 and promotes its K63-linked ubiquitination, which they describe to be important for MDA5 oligomerization and activation. The possibility that the chicken TRIM65 ortholog might be responsible for chMDA5 ubiquitination is currently under investigation.

Previous studies have shown that chMDA5 plays a role in the control of IBDV infection through the activation of the type I IFN pathway (Lee et al., 2014; Lee C. C. et al., 2015). Then, our results showing that chTRIM25 positively regulates IFN-β and NF-kB promoter activation by chMDA5 suggested that TRIM25 might act as a restriction factor for IBDV replication. In fact, higher IBDV replication was attained in TRIM25 KO DF-1 cells as compared with the parental DF-1 cell line, as determined by the higher accumulation of both the viral VP3 protein and RNA in infected cells, and the enhanced extracellular viral yields at 16 and 24 h pi. These results correlate with the reduced expression of *IFN-β*, *Mx* and *OAS* genes at both pi times, and the reduced expression of *PKR*, *FasR* and *IL6* at 24 h pi, although in these later cases the differences were not statistically significant. Similarly, upon infection of TRIM25 KO DF-1 cells with ARV, the expression of the muNS viral protein was enhanced with respect to the expression in the parental DF-1 cells. Also, higher GFP expression was observed in TRIM25 KO DF-1 cells infected with NDV-GFP and VSV-GFP recombinant viruses. Previous results from our laboratory showed that IFN plays a key role in apoptotic cell death of IBDV-infected cells (Cubas-Gaona et al., 2018; Broto et al., 2021). Significantly, reduced apoptosis triggering was observed after IBDV infection of TRIM25 KO DF-1 cells when compared with the parental DF-1 cells. As a whole, these results indicate that the reduced IFN response in the absence of chTRIM25 favors an increased permissiveness of the cells to viral replication and a reduction of the apoptotic response in IBDV-infected cells. In this regard, it was reported that chTRIM25 restricts the replication of subgroup A of avian leukosis virus through mediating the expression of type I IFN (Zhou et al., 2020). On the other hand, it was recently described that chTRIM25 inhibits IBDV replication by specifically interacting with the structural protein VP3 and triggering its ubiquitination and degradation (Wang et al., 2021). From our work we cannot discard the potential role of chTRIM25-mediated degradation of VP3. However, our attempts to find an interaction between VP3 and chTRIM25, as that described in that work were unsuccessful. This discrepancy could be due to differences between the viral strains used and/or experimental conditions employed in both studies.

As previously described, chMAVS overexpression is able to trigger IFN-β promoter activation and the downstream signaling (Liniger et al., 2012). Here we show that lower IFN-β and NF-kB promoter activation is achieved by overexpressing chMAVS in DF-1 TRIM25 KO cells compared with parental DF-1 cells, which indicates that chTRIM25 also plays a role downstream of chMAVS. On the other hand, we observed that co-expression of chTRIM25 wild-type, but not that of its C59S/C62S mutant version, along with chMAVS causes a dose-dependent reduction on the IFN-β promoter activation, and that this decrease correlates with a dose-dependent reduction in the accumulation of the MAVS protein, as determined by Western blotting assay. Similar amounts of *chMAVS* mRNA were detected upon co-expression of wild-type or mutant chTRIM25 (data not shown). These results would be compatible with the degradation of chMAVS in the presence of wild-type chTRIM25 but not of the C59S/C62S mutant. In this regard, it has been described that following RIG-I activation, TRIM25 mediates K48-linked ubiquitination and proteasomal degradation of MAVS, which, in one report was described to be required for type I IFN production (Castanier et al., 2012), while opposite results, like ours, were reported in another study (Liu et al., 2017). On one hand, these results support that the C59S/C62S mutation abrogates the ubiquitination activity of chTRIM25. On the other hand, they indicate that while endogenous chTRIM25 levels are required for full activation of IFN-β and NF-kB promoters by chMAVS, its overexpression exerts a deleterious effect by triggering chMAVS degradation. Concerning this, we have consistently observed that while co-transfection of low amounts (25 or 50 ng) of the chTRIM25 expression plasmid with the plasmid for chMDA5 results in the enhancement of IFN-β and NF-kB promoter activation, as described above, the use of a higher doses of the chTRIM25 plasmid (e.g., 100 ng), instead of rendering a further enhancement, causes a decline of the promoter stimulation activity. According to the above-mentioned results, this effect could be related with chMAVS degradation triggered by chTRIM25. Combined, these results indicate that chTRIM25 can exert opposite effects and that the expression level will largely affect the magnitude of the response.

A question arising from our results is: what is then the role of chTRIM25 on MDA5-mediated IFN induction pathway? A previous report showed that in mammalian cells TRIM25 is required for MDA5- and MAVS-mediated activation of NF-κB and IFN production (Lee C. C. et al., 2015). Upon activation, MAVS recruits the TNF receptor-associated factor 6 (TRAF6) to induce NF-κB activation (Yoshida et al., 2008), and TRAF6 K63-ubiquitination is a key event for this activation. Then, it was suggested that TRIM25 regulates NF-κB signaling by modulating TRAF6 ubiquitination. Our results are in agreement with the idea that TRIM25 acts downstream of MAVS in chicken cells, however, the fact that the C59S/C62S mutant lacking the ubiquitin ligase activity stimulates IFN-β and NF-kB promoter activation similarly to wild-type chTRIM25, suggests that chTRIM25 is not involved in TRAF6 ubiquitination. Emerging data evidence that TRIM25 is involved in different regulatory activities, working at multiple steps of signaling pathways leading to the induction of the innate immune response to virus infections, such as association with p53 and regulation of its intracellular levels, ISGylation of different targets and binding to RNA (Martín-Vicente et al., 2017). Further research will be required to decipher which is the molecular mechanism underlying the stimulatory effect of chTRIM25 on chMDA5-mediated IFN signaling activation.

In sum, data presented in this report indicate that chTRIM25 significantly contributes to regulate MDA5-mediated activation of the IFN-inducing pathway in chicken cells, at least at two levels, through its interaction with MDA5 CARD domains, and downstream of MAVS. Ablation of endogenous chTRIM25 reduces chMDA5-and chMAVS-induced IFN-β and NF-kB promoter activation and enhances permissiveness to viral infections. Additionally, ectopic expression of chTRIM25 together with chMDA5 enhances the response. However, excessive chTRIM25 expression attenuates this enhancement. Also, chTRIM25 overexpression causes the degradation of ectopically expressed chMAVS. Although the underlying mechanisms regulating these effects need to be clearly defined, our results contribute to enhance the knowledge about the regulation of IFN production in chicken cells, and could be useful in the search for antiviral strategies against IBDV and other important avian pathogens.

## Data availability statement

The raw data supporting the conclusions of this article will be made available by the authors, without undue reservation.

## Author contributions

AG-S, JR, and DR conceived the idea presented in this work. ED-B, LC-G, OC-R, AB-Z, MS-A, and LM conducted the experiments. ED-B, LC-G, MS-A, LM, JR, AG-S, and DR designed the experiments. ED-B and DR wrote the manuscript. ED-B, LC-G, JR, AG-S, and DR analyzed the data. AG-S, MS-A, LM, and JR provided critical revisions of the article. All authors contributed to the article and approved the submitted version.

## Funding

This work was supported by grants AGL2017-87464-C2-1-P and PID2020-112847RB-I00 to DR and JR, funded by MCIN/AEI/10.13039/501100011033 and by “ERDF A way of making Europe,” by the European Union. This work was also partly supported by CRIPT (Center for Research on Influenza Pathogenesis and Transmission), a NIAID funded Center of Excellence for Influenza Research and Response (CEIRR, contract number 75N93021C00014) to AG-S. ED-B was supported by a FPU fellowship from the Spanish Ministry of Education, Culture and Sport. LC-G was recipient of a predoctoral research contract from the International Fellowship Program of the Caixa Foundation and an EMBO short-term fellowship (ASTF 245-2015). OC-R was supported by a contract from the Consejería de Juventud y Deporte de la Comunidad Autónoma de Madrid (PEJD-2017-PRE/BIO-5006). DR was recipient of a visiting scholar fellowship (PRX16/00538) sponsored by Fulbright Program and Spanish Ministry of Education, Culture and Sport. Additional funding was received from the Spanish National Research Council (CSIC) to support open access publication fees. The funders had no role in study design, data collection and interpretation, or the decision to submit the work for publication.

## Conflict of interest

The AG-S laboratory has received research support from Pfizer, Senhwa Biosciences, Kenall Manufacturing, Blade Therapuetics, Avimex, Johnson & Johnson, Dynavax, 7Hills Pharma, Pharmamar, ImmunityBio, Accurius, Nanocomposix, Hexamer, N-fold LLC, Model Medicines, Atea Pharma, Applied Biological Laboratories and Merck, outside of the reported work. AG-S has consulting agreements for the following companies involving cash and/or stock: Castlevax, Amovir, Vivaldi Biosciences, Contrafect, 7Hills Pharma, Avimex, Vaxalto, Pagoda, Accurius, Esperovax, Farmak, Applied Biological Laboratories, Pharmamar, Paratus, CureLab Oncology, CureLab Veterinary, Synairgen and Pfizer, outside of the reported work. AG-S has been an invited speaker in meeting events organized by Seqirus, Janssen, Abbott and Astrazeneca. AG-S is inventor on patents and patent applications on the use of antivirals and vaccines for the treatment and prevention of virus infections and cancer, owned by the Icahn School of Medicine at Mount Sinai, New York, outside of the reported work.

The remaining authors declare that the research was conducted in the absence of any commercial or financial relationships that could be construed as a potential conflict of interest.

## Publisher’s note

All claims expressed in this article are solely those of the authors and do not necessarily represent those of their affiliated organizations, or those of the publisher, the editors and the reviewers. Any product that may be evaluated in this article, or claim that may be made by its manufacturer, is not guaranteed or endorsed by the publisher.

## Footnotes

^1^ https://imagej.nih.gov/ij/

^2^ https://bioinfogp.cnb.csic.es/tools/breakingcas/
