# Supplementary figures and images for: Interaction between chicken TRIM25 and MDA5 and their role in mediated antiviral activity against IBDV infection
Source: Front Microbiol. 2022 Nov 28;13:1068328. doi: 10.3389/fmicb.2022.1068328 (PMC9742432; doi:10.3389/fmicb.2022.1068328)

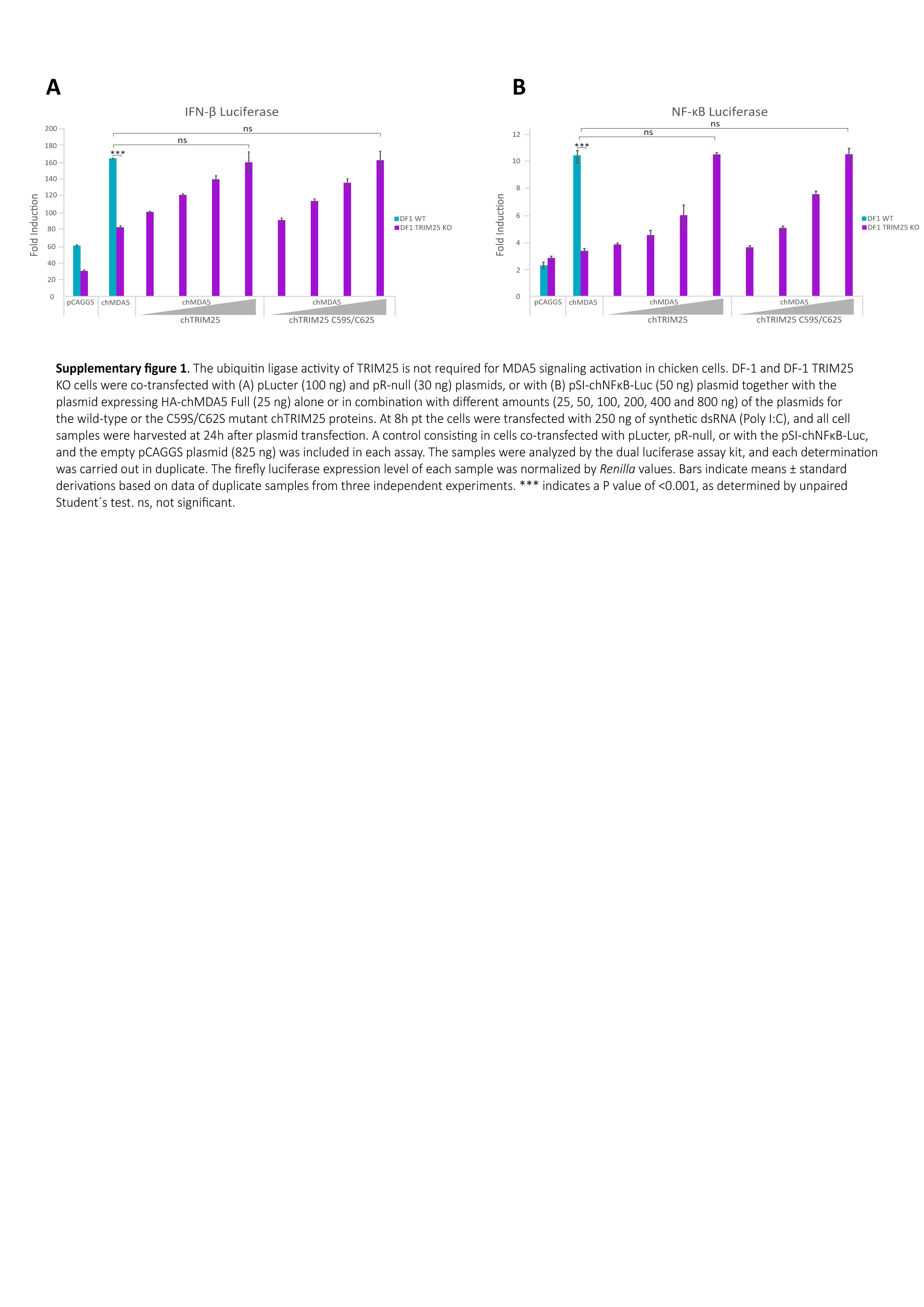

Supplement: Supplementary file 1 [file Image_1.jpg]

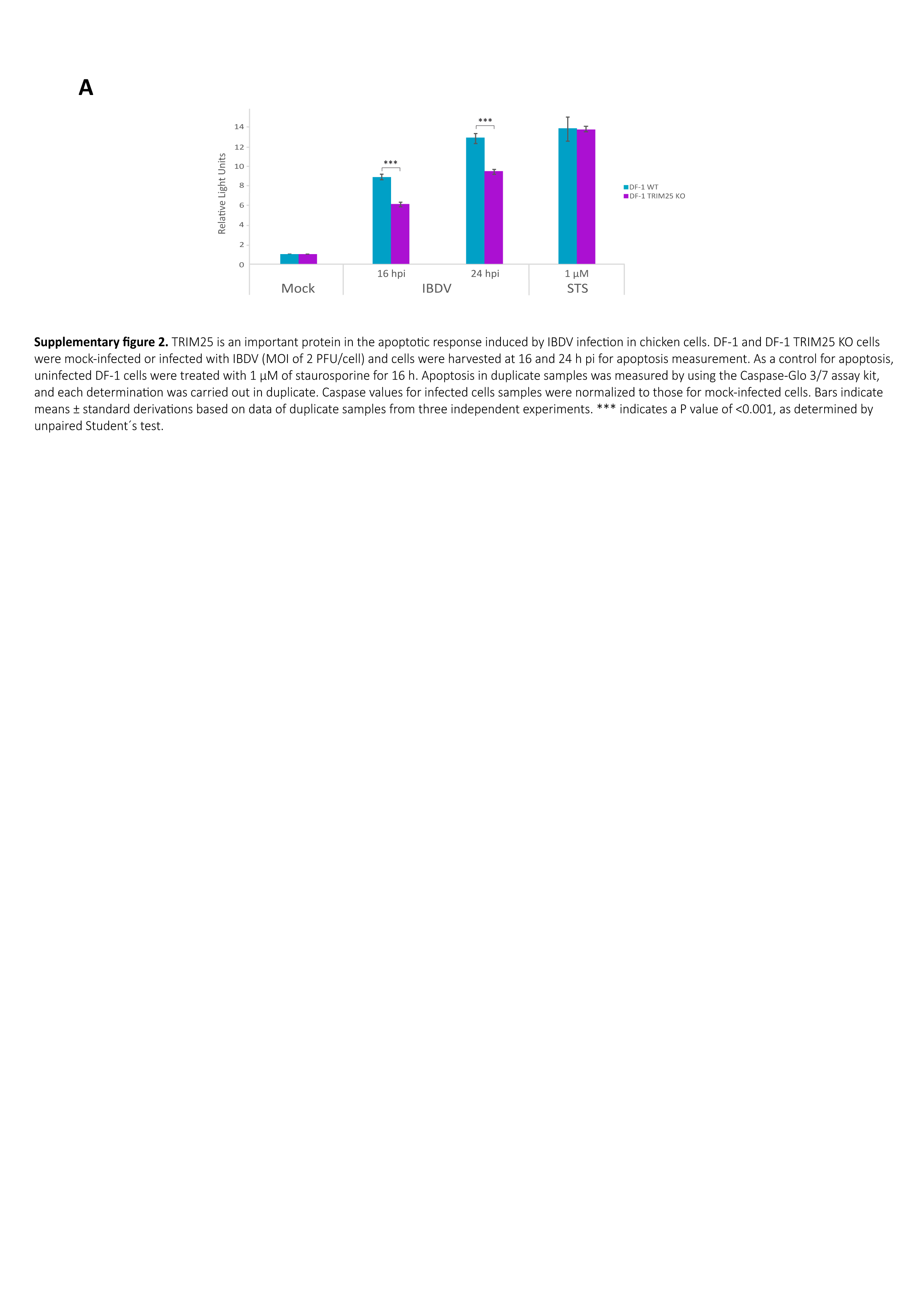

Supplement: Supplementary file 2 [file Image_2.TIF]
